# Prophenoloxidase-Mediated *Ex Vivo* Immunity to Delay Fungal Infection after Insect Ecdysis

**DOI:** 10.3389/fimmu.2017.01445

**Published:** 2017-11-01

**Authors:** Jie Zhang, Wuren Huang, Chuanfei Yuan, Yuzhen Lu, Bing Yang, Cheng-Yuan Wang, Peng Zhang, Leonard Dobens, Zhen Zou, Chengshu Wang, Erjun Ling

**Affiliations:** ^1^Key Laboratory of Insect Developmental and Evolutionary Biology, Institute of Plant Physiology and Ecology, Shanghai Institutes for Biological Sciences, Chinese Academy of Sciences, Shanghai, China; ^2^Shanghai Institute of Organic Chemistry Chinese Academy of Sciences, Shanghai, China; ^3^State Key Laboratory of Integrated Management of Pest Insects and Rodents, Institute of Zoology, Chinese Academy of Sciences, Beijing, China; ^4^State Key Laboratory of Virology and China Center for Virus Culture Collection, Wuhan Institute of Virology, Chinese Academy of Sciences, Wuhan, China; ^5^National Key Laboratory of Plant Molecular Genetics, Institute of Plant Physiology and Ecology, Shanghai Institutes for Biological Sciences, Chinese Academy of Sciences, Shanghai, China; ^6^School of Biological Sciences, University of Missouri-Kansas City, Kansas City, MO, United States

**Keywords:** prophenoloxidase, insect, fungi, *ex vivo* immunity, interaction

## Abstract

Skin immunity protects animals from airborne pathogen infection. Unlike mammals, arthropods, including insects, undergo periodic ecdysis to grow and develop. Newly molted insects emerge with unsclerotized thin cuticles but successfully escape pathogenic infections during the post-molt period. Here we show that prophenoloxidases (PPOs) in molting fluids remain bioactive on the integument and impede fungal infection after ecdysis. We found that the purified plasma PPOs or recombinant PPOs could effectively bind to fungal spores (conidia) by targeting the cell wall components chitin and β-1,3-glucan. Pretreatment of the spores of the fungal pathogen *Beauveria bassiana* with PPOs increased spore hydrophilicity and reduced spore adhesion activity, resulting in a significant decrease in virulence as compared with mock infection. We also identified a spore-secreted protease BPS8, a member of peptidase S8 family of protease that degrade PPOs at high levels to benefit fungal infection, but which at lower doses activate PPOs to inhibit spore germination after melanization. These data indicate that insects have evolved a distinct strategy of *ex vivo* immunity to survive pathogen infections after ecdysis using PPOs in molting fluids retained on the underdeveloped and tender integument of newly molted insects for protection against airborne fungal infection.

## Introduction

Skin or integument immunity is essential to protect advanced animals including human beings from pathogenic infection. Unlike mammals, arthropods, including insects, undergo periodical ecdysis between each larval stage to grow and finally metamorphosize into adults (1). Newly formed cuticles are thin and tender, and consequently prone to pathogen infection (2). The fact that insects remain the most successful creatures on earth with the largest number of species (3) suggests that they retain potent integument immunities after ecdysis.

Immunity proteins are detected in the insect molting fluids and midguts during the period of ecdysis (4–6). Many immunity proteins are expressed in the midguts to reduce the intestinal bacteria in both the bean bug *Riptortus pedestris* and *Bombyx mori*, likely to protect the molting larvae from midgut infection (4, 6, 7). During ecdysis, new cuticle is synthesized beneath the old one and molting fluid is secreted between the old and new cuticles (5). During this stage, the topical application of the spores (conidia) of insect pathogenic fungus *Beauveria bassiana* induced a systematic melanization in the molting fluids (5). Unexpectedly, it was found that fungal pathogen could not penetrate the new and thin cuticles to enter insect hemocoel (body cavity). Many immunity-related components of the molting fluids, including prophenoloxidase (PPO) and immune receptors like βGRP-3, have been identified (5). However, their roles in promoting insect survival from pathogen infections during the ecdysis period are still unclear.

Insect fungal pathogens are the essential regulators of insect populations in nature. Species like *B. bassiana* and *Metarhizium robertsii* have been used as models for the mechanistic investigation of insect–fungus interactions, and for biological control applications against insect pests (8–12). Distinct from insect pathogenic bacteria and viruses that infect insects through *per os* ingestion, the spores of fungal pathogens can be transmitted *via* air to adhere to the cuticular surface of host insects, germinate, and penetrate the integument to enter the insect hemocoel, where the fungal cells evade host immunity to propagate through yeast-type budding (13, 14). Because larvae after newly ecdysis have quite thin and underdeveloped cuticle (5), it is unclear how they can successfully escape pathogen infection after ecdysis.

In this study, by using the fungal pathogen *B. bassiana* as a model, we report that the PPOs present in insect molting fluids remain on the surface of newly formed cuticle and retain bioactivity to bind fungal spore. Surprisingly, a fungal spore-secreted protease that is a virulence factor could be co-opted by insects to cleave and activate PPOs to effectively melanize and inhibit fungal spore germination. Our results reveal that PPO-mediated *ex vivo* immunity on insect cuticles can protect hosts from fungal pathogen infection after newly ecdysis.

## Materials and Methods

### Insects, Fungi, and Bacteria

*Bombyx mori* (Nistari), *Helicoverpa armigera* larvae, and *Drosophila melanogaster* adults (*w^1118^, PPO1^Δ^, PPO2^Δ^ and PPO1^Δ^, PPO2^Δ^*) were reared as described (15–17). The strains of fungal pathogens *B. bassiana* ARSEF 2860, *M. robertsii* ARSEF2575, and its mutant Δ*MBZ1* were routinely maintained for the preparations of spore (conidia), hyphae, and blastospore as described (18). The yeast strains of *Saccharomyces cerevisiae* and *Candida albicans* were also routinely cultured for spore preparations (19). Spores (Chlamydospores) of *C. albicans* were collected (19). *Escherichia coli* (BL21) was cultured in lab. *Micrococcus luteus* (Sigma) was suspended in buffer for pull-down assay.

### PPO-Binding Assays

Spores of different fungal species were suspended in Tween 20 (0.05%, v/v) and centrifuged at 13,000× *g* for 2 min. Spores were then suspended in a binding buffer (50 mM Tris, 100 mM NaCl, pH 8.0) and washed three times. After last centrifuge, the spores were resuspended in binding buffer and the cell density was adjusted to 1 × 10^9^ cells/mL. The cells of *Micrococcus* were suspended in binding buffer to 100 µg/µL and the optical density (OD_600_) was determined after proper dilution. The cells of *E. coli* were collected, washed, and resuspended in binding buffer to the same optical density (OD_600_) as *Micrococcus*. Approximately, 1 µg of purified PPOs (BmPPO, HaPPO, rPPO1, rPPO2, and rPPO1-GFP) or 20 µL molting fluid (20 µg/µL with phenylthiourea (PTU) added to inhibit melanization) was mixed with approximately 1 × 10^7^ spores of different species or 1 × 10^7^ *Micrococcus* or *E. coli* in a 50-µL system (adjusted using the binding buffer). The above mixture was incubated by rotating at 4°C for 15 min. After incubation, fungal spores and bacteria were centrifuged at 13,000× *g* at 4°C for 2 min. The supernatant was used as the flow-through for Western blot assay or phenoloxidase (PO) activity assay. The cell pellets of different microorganisms were suspended in 100 µL washing buffer (100 mM Tris, 500 mM NaCl, pH 8.0), centrifuged (13,000× *g* at 4°C for 2 min) and washed for three times. During the second wash, the mixtures were transferred into a new tube to minimize protein contamination on the tubes. The washed cells were prepared for various purposes. The pelleted cells were suspended in 10 µL 1×SDS loading buffer and boiled for 10 min as elution. After centrifugation, the supernatant containing the eluted proteins was loaded for Western blot assay. Unless otherwise indicated, after binding assay using rPPO1 or rPPO2, approximately 8% and 16% of flow-through and elution solution treated as described above were loaded for each lane, respectively, in this work. To observe rPPO1-GFP fluorescence, pellets were suspended again in binding buffer and the fluorescence of spore was observed under microscope (Olympus BX51). To determine PO activity, the pellets were suspended in 200 µL of 10 mM dopamine prepared in 10 mM Tris buffer (pH 7.0) and incubated for 10 min. The same concentration of dopamine was added into the flow-through for PO activity assay. After the reaction, the mixture was then added with 10 µL of saturated PTU to stop PO activity and PMSF to a final concentration of 1 mM to inhibit protease activity. After centrifugation at 13,000× *g* for 2 min, the value of OD_490_ of the supernatant was recorded. The exact amounts of PPO and/or PO cannot be determined in the flow-through and on the spores here. Thus, in this case, one unit of PO activity was defined as ΔOD_490_/min = 1. To detect PO activity after being activated by the purified *B. bassiana* protease, one unit of PO activity was defined as ΔOD_490_/min/μg = 0.001.

To determine the binding potential of insect PPOs with the chitin and β-1,3-glucan, commercial chitin (NEB) and β-1,3-glucan, laminarin (Sigma) from *Laminaria digitata* usually used for invertebrate PPO activation (20, 21), were constituted in a binding buffer at room temperature for 15 min, and washed twice with the binding buffer. An amount of 0.1 µg of rPPO1 or rPPO2 was mixed with 2 mg chitin or β-1,3-glucan, and the samples were rotated at 4°C for 15 min. As a control, an amount of HaPPO (1 µg), BmPPO (1 µg), or GFP (1 µg) was mixed with 1 mg chitin or β-1,3-glucan. Chitin and β-1,3-glucan were washed, respectively, following the procedure used for spores. The bound protein was eluted exactly as spores were for Western blot assay. The flow-through was loaded to monitor the unbounded PPOs.

### Identification of PPO-Binding Components on Fungal Spores

To identify the targeted binding components, *B. bassiana* spores were treated in the following ways, and then used for binding assays. To remove spore surface proteins, spores were suspended and incubated in the binding buffer containing 0.5 µg/µL Protease K (Roche) at room temperature for 12 h. High concentration of HCl can hydrolyzate proteins (22). In order to remove all proteins, spores were also incubated in 37% HCl at room temperature for 12 h. To remove chitin component, spores were suspended and incubated in the binding buffer (200 µL) containing 1 µg/µL chitinase (Roche) and 1-mM phenylmethylsulfonyl fluoride (PMSF) (to inhibit protease contamination in this product according to our assay). To remove β-1,3-glucan component, spores were suspended and incubated in 200 µL of 0.2 M of citric acid–sodium citrate buffer (pH 5) containing 10 µg/µL glucanase (Sigma) and 1 mM PMSF at 37°C for 2 h. After these separate treatments (37% HCl, chitinase, and glucanase), spore samples were separated from chemical and washed in binding buffer for three times. Subsequently, the samples were incubated with rPPO1 or rPPO1-GFP as described above. In order to examine the enzyme hydrolysis effect, the dye Calcofluor White (Sigma) was applied for chitin staining (18), and β-1,3-glucan was detected by using Aniline Blue Fluorochrome (Biosupplies) (23).

### Competition Assay

Competition between the fragment PPO1-IP (PPO2-IP) and rPPO1 (rPPO2) to bind to spores was performed as follows. Approximately, 5 µg of the peptide fragments PPO1-IP (^518^NQALNLEEQR^527^) and PPO2-IP (^622^CSDAASYCGVR^632^) were suspended in the binding buffer and separately preincubated with 1 × 10^7^ spores in a 50-µL system by rotating at 4°C for 15 min. After that, rPPO1 (0.06 µg) and rPPO2 (0.5 µg) were separately added to each tube containing the corresponding competitor peptide for another 15 min. As control, the peptide fragments PPO1-IP and PPO2-IP were not preincubated with spores. The above mixtures were centrifuged to obtain the flow-through solution, and the spores were washed as described above and proteins were eluted for Western blot assay.

### Spore-Secreted Protease Isolation, Gene Cloning, Expression, and PPO Cleavage Assay

To identify the spore-secreted proteases, 1 × 10^7^ spore were suspended and agitated in 50-µL binding buffer at room temperature for 12 h. The supernatant was collected and concentrated for analysis with an 8% native gel containing gelatin (1 mg/mL) (Gelatin Zymography) as described (24). The gel was then incubated in a binding buffer at 37°C for 2 h followed by Coomassie blue staining and destaining. Another native gel without gelatin was run in parallel for separating protease. The corresponding position of the white band on the gelatin gel was cut from the native gel (without gelatin) and treated for liquid chromatography–mass spectrometry (LC–MS)/MS assay as described (25). The genome database of *B. bassiana* was used for the search of fungal proteins (26).

Full-length cDNA of *BPS8* (BBA_00319) (26) was amplified from a cDNA library and cloned into a pET28 vector and transformed into *E. coli* BL21 (see Table S1 in Supplementary Material for primer information). *BPS8* was induced to express and purified as described (27). Purified BPS8 was reconstituted in Tris buffer (10 mM, pH 7.0) and the concentration was determined. Different amount of BPS8 was incubated with 1 µg recombinant PPO1 (rPPO1) in a 20-µL system prepared in the binding buffer for 10 min, then the mixture was added with PTU to stop PO activity and PMSF (1 mM) to inhibit protease activity. PO activities were assayed by addition of 200 µL of 10 mM dopamine and incubating for 10 min before recording the OD_490_ values. The reaction sample was also loaded for Western blot assay to demonstrate rPPO1 cleavage.

### Protease Gene Deletion and Complementation

To determine the contribution of BPS8 to activate insect PPOs or fungal virulence, the gene was deleted in *B. bassiana* by homologous replacement using the *Agrobacterium*-mediated transformation (18). To complement the gene deletion, full open reading frame of the gene together with its promoter region was amplified and cloned the binary vector to transform Δ*BPS8* (18). Drug resistant mutants were selected and verified by polymerase chain reaction.

### Melanin and Melanization Inhibition on Spore Germination

To determine the effect of PPO-mediated melanization on fungal spore viability, the spores of *B. bassiana* were first treated with commercial melanin (Sigma, 10 µg/µL) suspended in a minimal medium at 28°C for 12 h and rotated at 200 rpm. After centrifugation at 1,000× *g* for 5 min, the saturated supernatant was transferred to a new tube and labeled as 100%. Different concentration of melanin was diluted in minimal medium to 25% and 50%, respectively. *B. bassiana* spores were suspended in different concentrations of melanin solution and rotated at 25°C for 12 h to count the germinating spores.

To mimic the melanization process of molting fluids upon contacting fungal spores, 2.5 µg rPPO1 or BSA (control) was incubated with 1.0 × 10^7^ spores suspended in binding buffer (total volume 50 µL) at room temperature for 15 min. Then, one aliquot was added with 150 µL of saturated l-3,4-dihydroxyphenylalanine (l-DOPA) (dissolved in a minimal medium) and 10 µL water, and the other aliquot was added with 160 µL minimal medium as control, and the third aliquot was added with 10 µL saturated PTU (to inhibit melanization) and 150 µL saturated l-DOPA. The spores were incubated by rotation at 25°C, and the germinating rates were determined 12 h post-incubation.

### Spore Adhesion and Hydrophobicity Assays

To determine the effect of PPO binding on spore hydrophobicity, the spores of *B. bassiana* (1.0 × 10^7^) were incubated with 2.5 µg of rPPO1 or rPPO2 at room temperature for 15 min. Hydrophobicity index was determined as using hexadecane described (18). To assay the spore adhesion ability after being bound by rPPO1 or rPPO2, spores were washed in a Tris buffer (50 mM, pH 8.0) three times and suspended in the same buffer to adjust the spore density to 1.0 × 10^5^ spore/mL. Aliquots of 100 µL spore suspension were loaded into a six-well plastic plate (Corning) for 1 h and then washed to determine the ratio of spore binding to the hydrophobic surface (26).

### Insect Bioassays

*B. bassiana* spores (1.0 × 10^8^) were incubated with 2.5 µg of rPPO1 or rPPO2 in 0.05% Tween 20 (v/v) at room temperature for 15 min. Topical infection of silkworm larvae (on day 2 of 5th larval stage, V-2, 15 insects for each replicate) or wild type (WT) and mutant (*PPO1^Δ^* and *PPO2^Δ^*) *Drosophila* female adults (3 days after eclosion; 20 insects for each replicate) were performed by immersion in the spore suspension for 30 s (28). In another experiment, at 40 h post the topic infection by spores preincubated with rPPO1 or rPPO2 or nothing as described above, the melanized larvae were observed and counted. Hemolymph of each larva was sampled, and then 25 µL hemolymph was diluted for smearing one Potato Dextrose Agar plate. The numbers of hyphae colonies were counted and compared. To determine the importance of molting-fluid residues in inhibiting *B. bassiana* infection, silkworm larvae were washed immediately with sterile water for 2 min after the third and fourth ecdysis. The washed larvae were dried on paper, and the washed and unwashed larvae were placed side by side and sprayed with spore suspension (1.0 × 10^7^/mL). The insects were fed in a box containing wet paper to maintain moisture. To determine the contribution of BPS8 to fungal virulence, insect bioassays were conducted using the spores of WT, Δ*BPS8* and gene-rescued mutant (1.0 × 10^8^, respectively) for topic infections of the silkworm larvae (V-2, 15 insects for each replicate). Approximately, 1.0 × 10^5^ spores of WT, Δ*BPS8*, and gene-rescued mutant were injected into pupae on day 1 (P-1). On day 6 after injection, the pupae were compared and imaged. For all bioassays, each experiment was repeated three times and mortality was recorded every 12 h. The median lethal time (LT_50_) was calculated as described (18).

### Western Blot Analysis

Rabbit polyclonal antibodies against the silkworm PPO (1: 5,000) (29), 30Kc19 (1: 3,000) (30), *M. sexta* βGRP-2 (1: 2,000) (31), *Drosophila* PPO1 (1:5,000) (27), and mouse polyclonal antibody against *Drosophila* PPO2 (1: 1,000) were used as the primary antibodies for Western blot analyses. Antibody binding was visualized by a color reaction catalyzed by alkaline phosphatase (AP) conjugated goat anti-rabbit/mouse IgG (1:10,000; Chemicon), or visualized by enhanced chemiluminescence catalyzed by horseradish peroxidase (HRP) using Pierce ECL Western blot substrate (Thermo).

## Results

### Molting Fluids Important to Delay Fungal Infection on Larvae after Ecdysis

We showed before that the proteins identified in insect molting fluids contribute to the antimicrobial activities and ecdysis regulation (5). After ecdysis, molting fluid dries quickly on the cuticle surface of newly molted silkworm *B. mori*, and we suspected that immunity-related proteins of *B. mori* like PPO (BmPPO) might remain bioactive on the insect cuticles for a certain period. To test this, we applied dopamine on the silkworm integument at different time points and found that PO activity could be detected up to 72 h after silkworm larvae emerged into the fifth instar (Figure S1A in Supplementary Material). In addition, the proteins of BmPPOs were detected in the molting fluids throughout this period by Western blotting analysis (Figure S1B in Supplementary Material). BmβGRP-3 was also detected in the washed solution (data not shown). After further washing, no BmPPOs could be detected (Figure S1C in Supplementary Material), and the PO activity was significantly reduced (Figure S1D in Supplementary Material).

**Figure 1:**
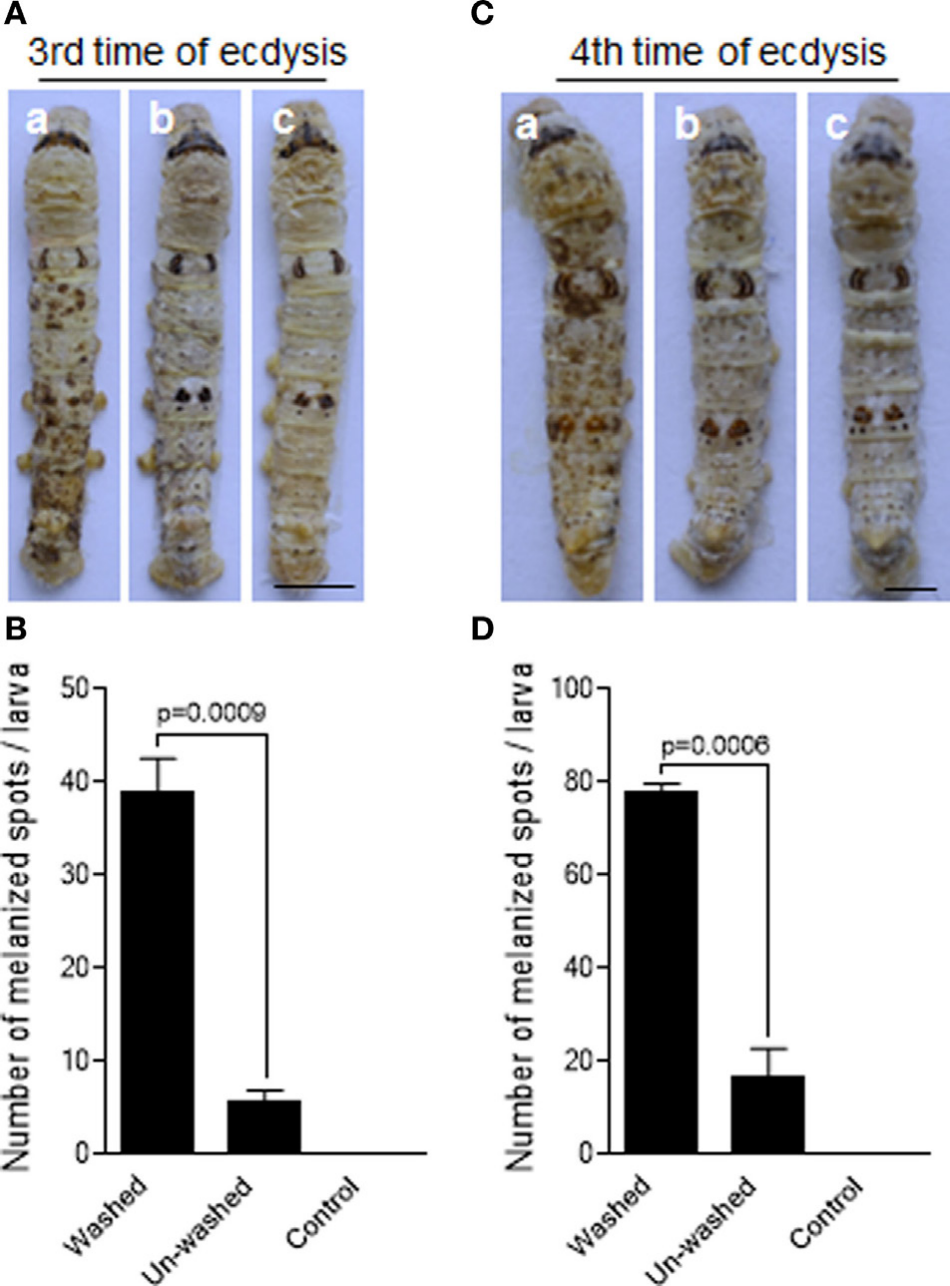
Delay of fungal infection by the molting-fluid residues. The silkworm larvae after the third or fourth ecdysis were washed to remove molting-fluid residues. The washed and unwashed larvae were infected by *Beauveria bassiana* through spraying spore suspension. On 2 days after infection, discrepancy in insect body melanization was observed, counted, and compared between different treatments **(A,C)**. Washed and infected larvae became heavily melanized (a). Body melanization was not apparently observed for those not washed but infected larvae (b), and those washed but not infected larvae (c). The number of melanized spots on the integuments were significantly higher if the molting fluids were removed after washing **(B,D)**. In **(B,D)**, each column represents the mean ± SE estimated from three individuals. Bar: 0.5 cm.

*B. mori* like PPOs in the molting fluids were significantly removed when the newly molted larvae were washed using water within 2 h after ecdysis (Figures S1C,D in Supplementary Material). To test the protective effects of molting-fluid residues on fungal infection, silkworm larvae (within 2 h post the third time of ecdysis) were either washed or unwashed before the topical infection with the spores of *B. bassiana* by spraying method. We found that the larvae washed of molting-fluid residue became heavily melanized two days after fungal infection as compared with unwashed or uninfected insects (Figure 1A). If the numbers of visible melanized spots are compared, the differences are significant among the washed and unwashed treatments (Figure 1B). Similar results were obtained when using the silkworm larvae after the 4th ecdysis (Figures 1C,D). Usually there are not so many fungal spores in the insect living environment, from a mean of 0.2 CFU/m^3^ during 10 months per year in an Europe country to a range from 0 to 3.1 × 10^3^ CFU/m^2^/day in a forest in Japan (32), which is far below high spore concentrations used in this study (1.0 × 10^7^/mL) and in other labs. These data demonstrate that the molting-fluid residue is potently biologically active and likely functionally essential to protect larvae from fungal infection after ecdysis.

### PPOs Specifically Binding to *B. bassiana* Spore

**Figure 2:**
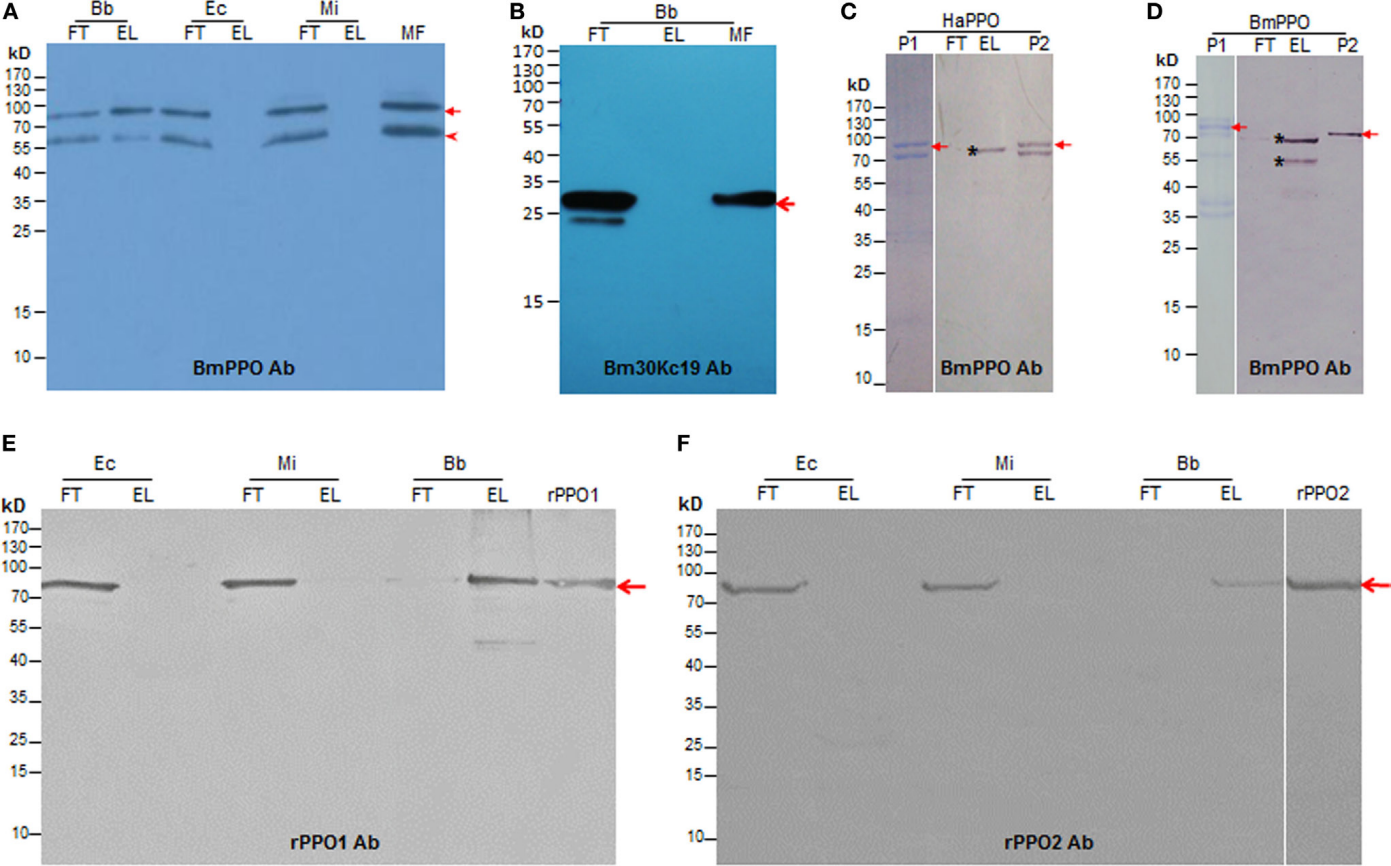
Insect PPOs bind to the spore of *B. bassiana* (Bb). **(A)** BmPPO in the molting fluid (MF) residues binds to the spore of Bb. Diluted MFs containing PTU and PMSF were incubated with the Bb spore, Ec, and Mi at 4°C for 15 min. Western blot analysis indicated that BmPPO including a degraded fragment that might be cleaved during the molting stage (5) as indicated by the arrowhead could only be eluted from fungal spore but not the bacterial cells. BmPPOs were indicated by the arrow. **(B)** A reference protein 30Kc19 (arrow-pointed) in MFs could not bind to the spores. **(C)** Purified plasma PPO from HaPPOs (33) binds to Bb spores. P1: HaPPOs were purified to homogeneity (arrow) as indicated in PAGE analysis. P2: Purified HaPPOs included in Western blot analysis. The asterisk indicates fragments eluted from fungal spores, which was cleaved from HaPPOs during incubation. **(D)** Partially purified BmPPO binds to Bb spores. BmPPO was arrow-indicated (P1) in PAGE analysis; P2: Purified BmPPO included in Western blot analysis. The arrow points to BmPPO band. The asterisk indicates that a fragment that was degraded from BmPPO during incubation was similarly eluted from spores. **(E,F)** Binding of purified recombinant *Drosophila* PPO1 (E; rPPO1) and PPO2 (F; rPPO2) to Bb spores. rPPO1 and rPPO2 were individually incubated with Bb spores and different bacteria cells. The arrows point to rPPO1 and rPPO2, respectively. Bb, *B. bassiana*; BmPPO, *Bombyx mori* plasma PPO; Ec, *Escherichia coli*; EL, elution; FT, flow-through; HaPPOs, *Helicoverpa armigera*; MF, molting fluid; MI, *Micrococcus*; PPOs, prophenoloxidases; PTU, phenylthiourea; rPPO1, recombinant PPO1; rPPO2, recombinant PPO2.

To identify the protein(s) functioning in the molting fluid, residue was incubated with either *B. bassiana* spore, *E. coli* (G^−^), or *M. luteus* (G^+^). Western blotting analysis indicated that BmPPOs could be eluted specifically from fungal spore but not from bacterial cells (Figure 2A). As a control, we found that a low molecular-weight protein (30 kDa) named Bm30Kc19 (NCBI Accession: ADQ89805) (30), previously identified in the molting fluids, could not bind to fungal spore (Figure 2B). To further compare the binding activity of insect PPOs to fungal spores, plasma PPOs were purified from the silkworm *B. mori* (BmPPO) and cotton bollworm *H. armigera* (HaPPO) (33), and used to treat the spore of *B. bassiana*. The results indicated that both plasma HaPPO and BmPPO could bind to spore (Figures 2C,D). Likewise, the plasma PPO1 and PPO2 from *D. melanogaster* were able to bind to fungal spores (Figures S2A,B in Supplementary Material).

**Figure 3:**
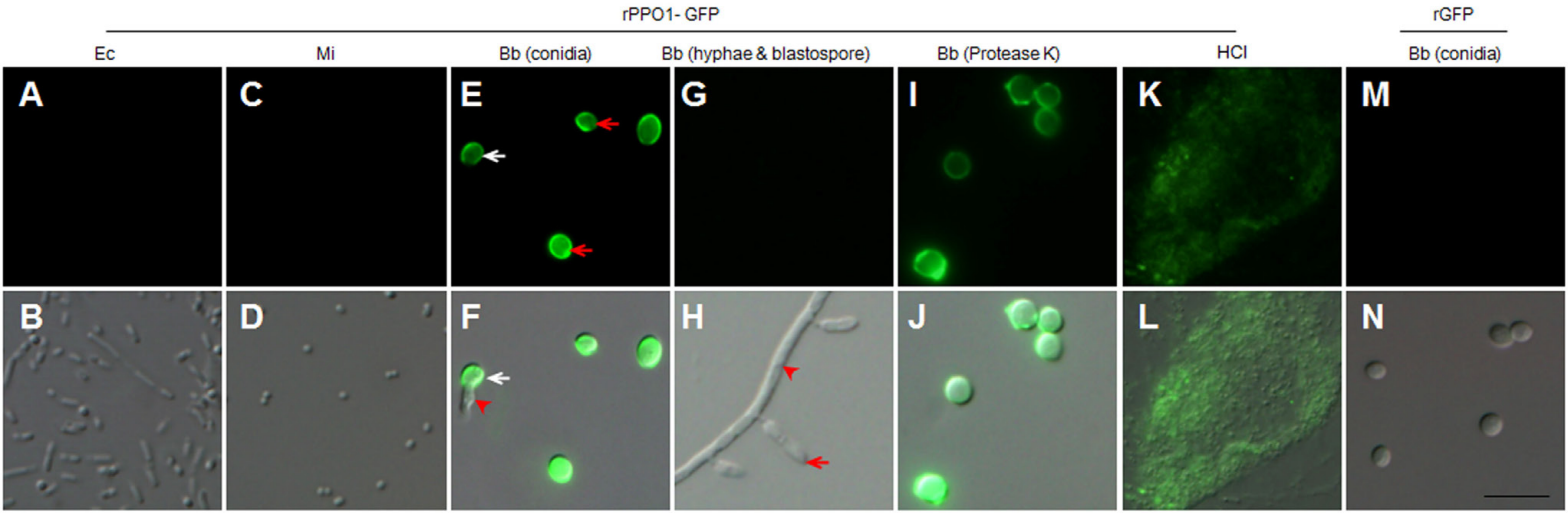
rPPO1-GFP binding assays. rPPO1 was fused with GFP (rPPO1-GFP), expressed and purified (35) for incubation with the cells of *Escherichia coli* **(A,B)**, *Micrococcus* **(C,D)**, *Beauveria bassiana* spores **(E,F)**, hyphae and blastospores **(G,H)**, and protease-K and 37% HCl **(K,L)** treated spores overnight. rGFP was applied as a control protein to monitor the binding assay **(M,N)**. rPPO1-GFP could bind to fungal spores **(E,F)** but not bacterial cells **(A–D)**. rPPO1-GFP did not bind to *B. bassiana* hyphae and blastospore **(G,H)**. In the panels **(E,F)**, the white arrow points to the germinating spores and the arrowhead points to a growing hypha. In panel H, the arrow points to a blastospore and the arrowhead points to the hyphae. rPPO1-GFP also bounds to *B. bassiana* spores with cell wall proteins removed by protease K **(I,J)** and spore residual after hydrolysis by HCl **(K,L)**. rGFP did not bind to the cell walls of *B. bassiana* spores **(M,N)**. Panels on the top row are images taken using GFP filter and the bottom row panels were merged with those imaged using DIC. DIC, differential interference contrast; rPPO1, recombinant PPO1; rPPO2, recombinant PPO2. Bar: 5 µm.

Next, we produced recombinant PPO proteins in *E. coli* to examine their fungal binding activity. Consistent with a previous observation in *Manduca sexta* (34), recombinant BmPPO had no PO activity whereas *Drosophila* PPO1 and PPO2 expressed in *E. coli* exhibited PO activities (27). Thus, rPPO1 and rPPO2 of *Drosophila* were purified and incubated separately with the spore of *B. bassiana* and the cells of *E. coli* and *Micrococcus*. Confirmatively, rPPO1 and rPPO2 could bind to fungal spores but not to bacterial cells (Figures 2E,F). The addition of protease cocktail to inhibit the potential fungal proteases did not disturb rPPO1 and rPPO2 to bind to spores (data not shown). Our previous study showed that the GFP-fused rPPO1 (rPPO1-GFP) could exhibit both the PO activity (after being activated) and GFP fluorescence (35). The incubation of purified rPPO1-GFP with the cells of different microorganisms revealed no fluorescent signals associated with the cells of *E. coli* and *Micrococcus* (Figures 3A,D) but strong fluorescence associated with fungal spore surfaces (Figures 3E,F). Interestingly, rPPO1-GFP did not bind to the germinated hyphae and blastospores of *B. bassiana* (Figures 3G,H; Figure S3A in Supplementary Material). Recombinant rPPO1-GFP could also bind to the heat- or NaN_3_-killed spores of *B. bassiana* (Figure S3B in Supplementary Material). The rGFP protein alone as a control did not bind to spore (Figures 3M,N; Figure S3B in Supplementary Material). We also tested the binding activities of rPPO1 to other fungal spore types and detected binding to the spores of insect pathogen *M. robertsii* but only weak binding to the sores of *S. cerevisiae* and the chlamydospores [also called as spores (19)] of *C. albicans* (Figures S4A,B in Supplementary Material).

### Insect PPOs Binding to the Cell Wall Components Chitin and β-1,3-Glucan

The cell wall of *B. bassiana* spore consists mainly of chitin, glucans, and proteins (36), so we sought to determine the component of fungal cell wall targeted by PPOs. First, protease K was incubated with spores overnight to digest spore cell wall proteins (22, 37). We found that the resulting spores could be effectively bound by rPPO1-GFP (Figures 3I,J). When HCl was incubated with spores overnight to hydrolyze proteins as described (22), the cell wall debris was still able to bind rPPO1-GFP (Figures 3K,L). Thus, the proteins on spore cell wall are likely not the main binding targets for PPO.

**Figure 4:**
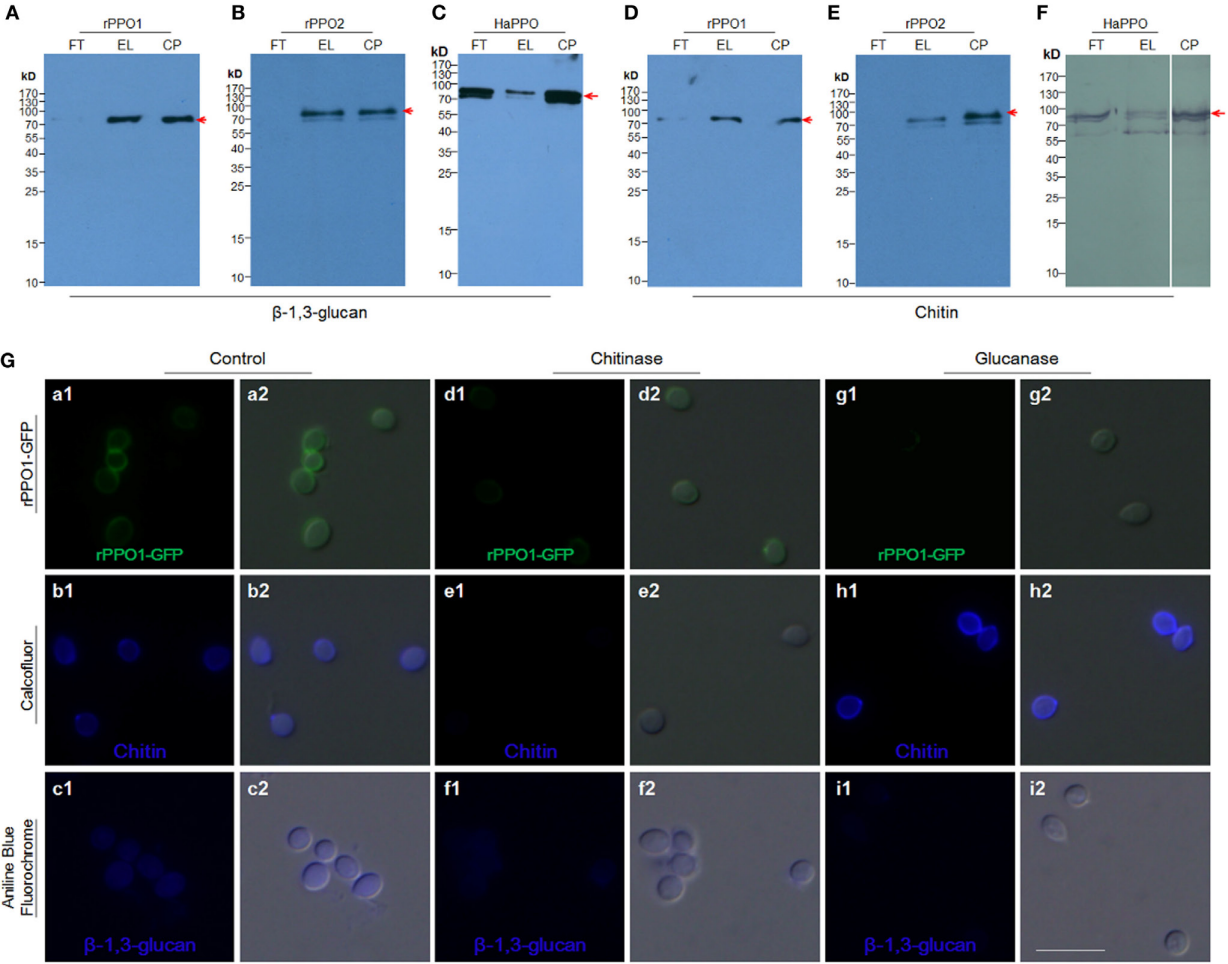
Insect PPOs bind to fungal spores *via* the cell wall components of chitin and β-1,3-glucan. **(A–C)** Insect PPOs bind to β-1,3-glucan. Purified rPPO1 **(A)**, rPPO2 **(B)**, and HaPPO **(C)** were individually incubated with β-1,3-glucan. PPOs were eluted from β-1,3-glucan for Western blot assay. **(D–F)** Insect PPOs bind to chitin. Purified rPPO1 **(A)**, rPPO2 (B), and HaPPO **(C)** were individually incubated with chitin, and then eluted for Western blot analysis. **(G)** Impairment of rPPO1-GFP binding of *B. bassiana* spores after the treatments with chitinase and glucanase. *B. bassiana* spores were incubated with chitinase or glucanase to remove chitin or β-1,3-glucan from the cell wall, respectively. Decreased amount of rPPO1-GFP signal was found for the spores treated with chitinase (compare b1, b2, e1, e2), or glucanase (compare c1, c2, i1, i2) according to Calcofluor and Ananline Blue Fluorochrome staining. EL, elution; FT, flow-through; CP, control protein; PPOs, prophenoloxidases; rPPO1, recombinant PPO1; rPPO2, recombinant PPO2; Bar: 5 µm.

We next tested the binding potential of PPOs against the cell wall components glucan and chitin. Both rPPO1 (Figures 4A,D) and rPPO2 (Figures 4B,E) bound to β-1,3-glucan and chitin, respectively. Likewise, the purified plasma HaPPO (see Lane P1 in Figure 2C without any treatment) bound to both β-1,3-glucan and chitin (Figures 4C,F). In contrast, when GFP was incubated with β-1,3-glucan and chitin, respectively, no binding was detected (Figure S5 in Supplementary Material). We then treated fungal spore with either chitinase or glucanase, and performed rPPO1-GFP binding assays and counterstaining with the fluorescence dyes Calcofluor White and Aniline Blue, which detect remaining chitin and β-1,3-glucan components, respectively. The results indicated that while untreated spores bound tightly to rPPO1-GFP (Figure 4G, a1,a2), spores treated with the chitinase or glucanase had strongly reduced GFP-signals (Figure 4G, d1,d2,g1,g2). Confirming the activity of glucanase, Aniline Blue staining could barely detect the β-1,3-glucan signals (Figure 4G, i1,i2), and the chitinase-treated spores had barely detectable staining-signals of both the chitin and β-1,3-glucans (Figure 4G, e1,e2,f1,f2), which could be due to the fact that chitin is localized inside the cell walls (36). When we performed the binding assay on a null mutant of *M. robertsii* (Δ*MBZ1*), which has enhanced accumulation of chitin on the cell wall (18), we found that rPPO1-GFP bound more strongly than the WT spores (Figures S4B, b1,b2 in Supplementary Material). Notably, the presence of carbohydrate like sugars (Figures S6A,B in Supplementary Material) or peptidoglycan from Gram-positive and Gram-negative bacteria (Figures S7A,B in Supplementary Material) in the mixture did not significantly influence rPPO1 or rPPO2 binding to spores. We also notice that Lipopolysaccharide did not disturb the binding (data not shown). rPPO1 or rPPO2 was pretreated with either chitin or β-1,3-glucan. rPPO1 and rPPO2 in the flow-through after removing chitin or β-1,3-glucan still bound to spores without significant difference (Figures S8A,B in Supplementary Material). In addition, rPPO1 or rPPO2 did not significantly disturb rPPO1-GFP to binding to spores (Figures S9A,B in Supplementary Material). These data indicate that rPPO1 and rPPO2 can bind to the spores *via* cell wall components chitin and β-1,3-glucan.

### rPPO1 and rPPO2 Binding to *B. bassiana* Spores through Specific Peptide Fragments

**Figure 5:**
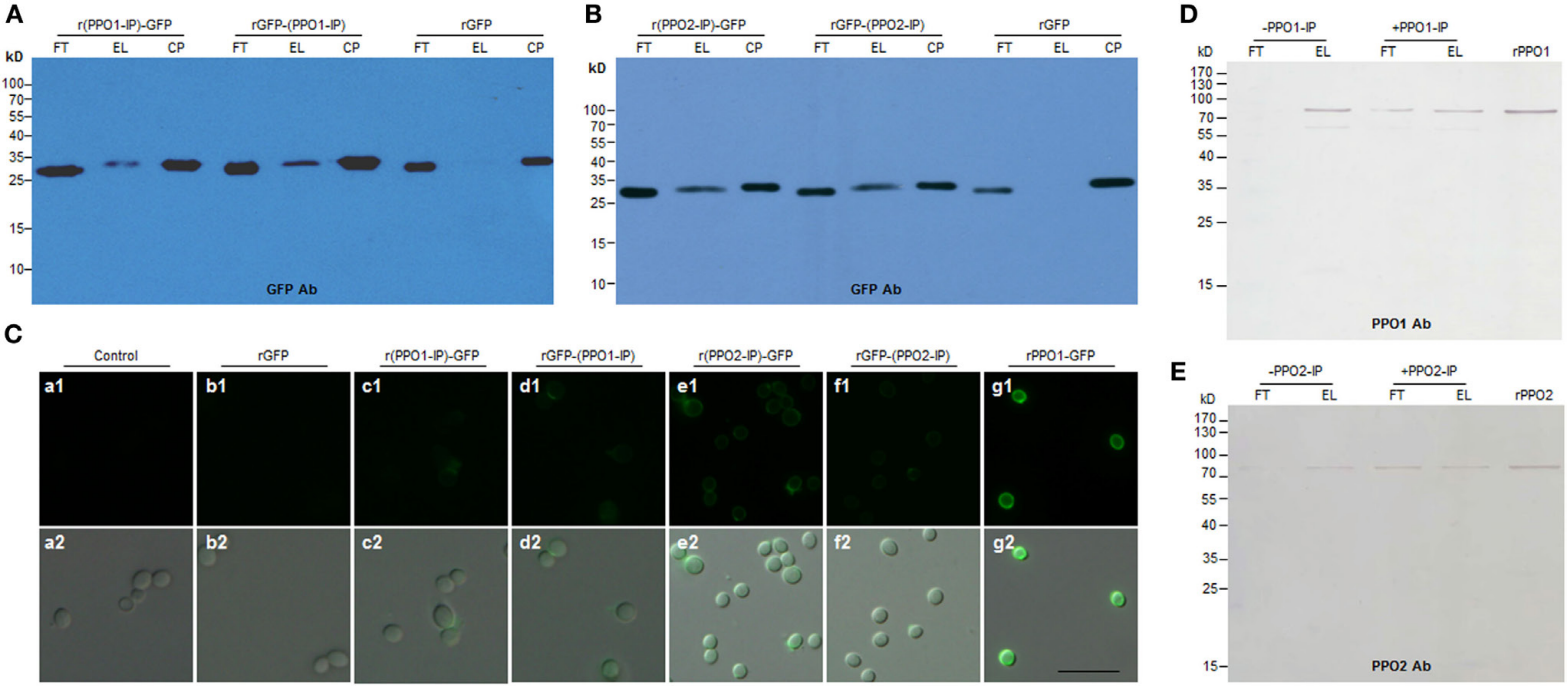
Functional verification of the binding activity of the fragments identified from PPO1 and PPO2. **(A,B)** Western blot confirmation of fragment-mediated binding to *B. bassiana* spore. The fragment identified from PPO1 (labeled as PPO1-IP) or PPO2 (labeled as PPO2-IP) was fused with GFP either at N-terminus (r(PPO1/2-IP)-GFP) or C-terminus [rGFP-(PPO1/2-IP)]. After purification, the fused proteins were incubated with *B. bassiana* spores and detected by antibody against GFP for Western blot assay. **(C)** Microscopic examination of spore binding. The purified GFP-fused fragments and other proteins as indicated were used to incubate with *B. bassiana* spores for binding assay. Control spores were not incubated with any protein. **(D,E)** Fragments PPO1-IP and PPO2-IP inhibited rPPO1 and rPPO2 to bind to the spores, respectively. PPO1-IP (labeled as + PPO1-IP) and PPO2-IP (labeled as + PPO2-IP) were incubated with the spores for 15 min before the addition of corresponding proteins (rPPO1 and rPPO2). As control, no PPO1-IP (labeled as -PPO1-IP) and PPO2-IP (labeled as -PPO2-IP) were preincubated with the spores. The preincubation with fragments PPO1-IP and PPO2-IP inhibited rPPO1 and rPPO2 to bind to spores. rPPO1 (10 ng) and rPPO2 (80 ng) were loaded for each lane to indicate their positions. Bar: 5 µm. CP, control proteins; EL, elution; FT, flow-through; rPPO1, recombinant PPO1; rPPO2, recombinant PPO2; Bar: 5 µm.

We next sought to identify the domain(s) in the rPPO1 and rPPO2 proteins that mediate interactions with fungal cell wall components. To determine the binding site, the proteins rPPO1-GFP, rPPO1, and rPPO2 were individually incubated with fungal spores in buffer for 4 h and then treated with trypsin for 4 h (Figure S10 in Supplementary Material). When bound fragments were then eluted for LC–MS/MS assay, the trypsin fragments ^518^NQALNLEEQR^527^ from PPO1 (named as PPO1-IP) (Figure S11A in Supplementary Material), and ^622^CSDAASYCGVR^632^ (named as PPO2-IP) from PPO2 were identified (Figure S11B in Supplementary Material). The corresponding DNA sequences encoding these two fragments were synthesized and fused in frame with a GFP protein gene at either the N-terminus or C-terminus. The resulting fusion proteins r(PPO1-IP)-GFP, rGFP-(PPO1-IP), r(PPO2-IP)-GFP, and rGFP-(PPO2-IP) were expressed, purified, and then incubated with the spore of *B. bassiana*, respectively. Both Western blotting assays (Figures 5A,B) and fluorescence microscopy (Figure 5C, b1–e2) analysis confirmed that, relative to the controls, these GFP-fused fragments bound to spores more strongly than rGFP, but with a weaker signal than observed for full-length rPPO1-GFP (Figure 5C, g1,g2). When the peptide fragments PPO1-IP and PPO2-IP were preincubated with the spores, these peptides inhibited the binding activity of rPPO1 and rPPO2 (Figures 5D,E). We also performed an *in silico* molecular docking analysis of rPPO1 and rPPO2 based on the conserved structure of insect PPOs (38, 39). Consistently, both chitin and β-1,3-glucan could be docked predictively into cavities composed of the above identified fragments. Notably, these two cavities are not in the same surface areas of PPO1 and PPO2 (Figure S12 in Supplementary Material). Therefore, rPPO1 and rPPO2 bind to spores through specific peptide fragments. However, the integrality of PPO structure should be advantageous to binding.

### Identification of a Spore-Secreted Protease from Fungal Spore That Contributes to the Activation of PPOs

**Figure 6:**
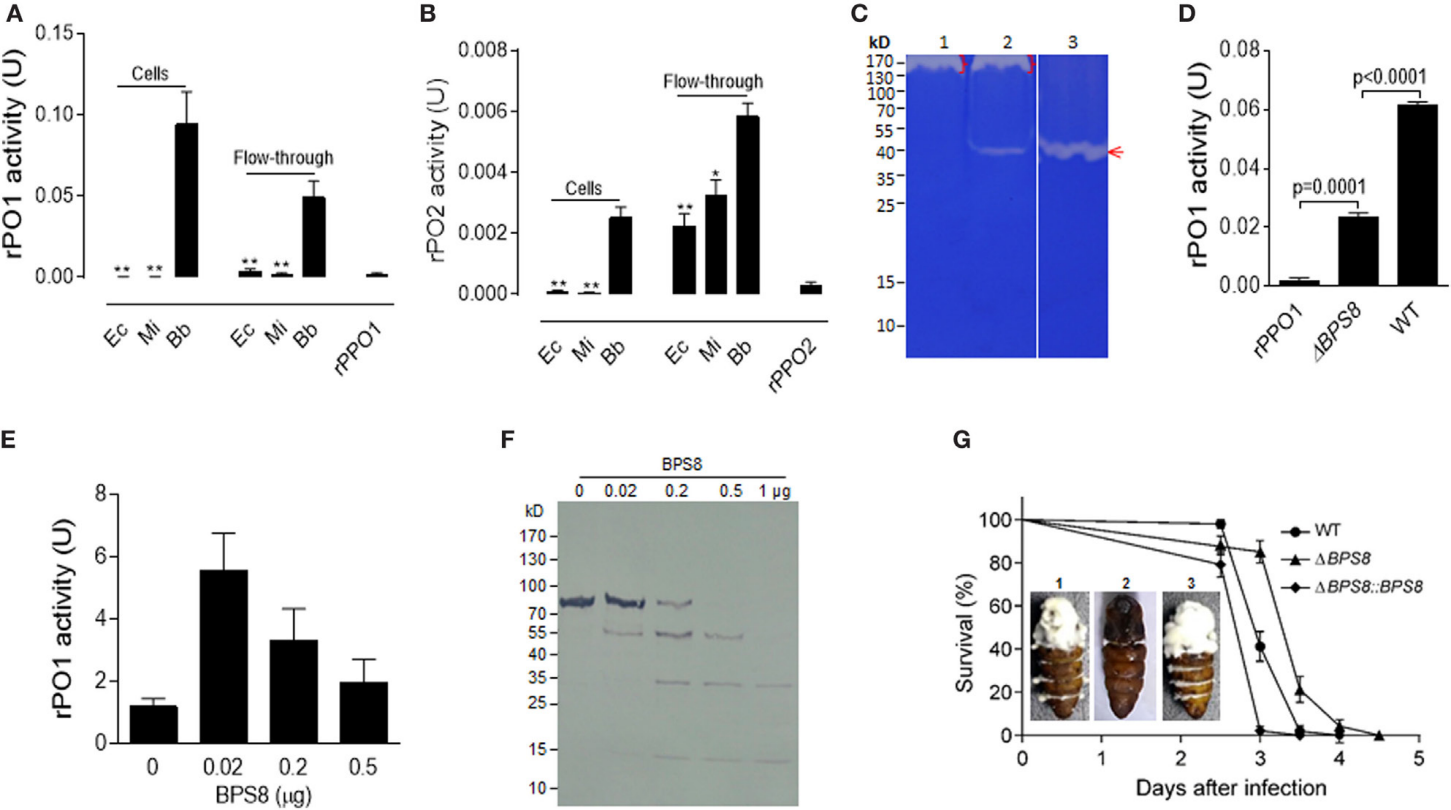
Activation and degradation of insect PPOs by fungal spore-secreted protease BPS8. **(A,B)** Detection of rPO1 **(A)** and rPO2 **(B)** activities in spore and flow-through samples. After the incubation of bacterial cells and fungal spores with rPPO1 or rPPO2, rPO1/rPO2 activities could only be detected on fungal spores and in their flow-through. * *p* < 0.05; ** *p* < 0.01. **(C)** Identification of spore-secreted protease BPS8. The protein band detected on a gelatin gel was identified as BPS8 by LC–MS/MS analysis. The genes were cloned and expressed in *E. coli*, and the purified protein BPS8 has a protease enzyme activity (Lane 3). Lane 1, protein sample isolated from the *ΔBPS8* spores of *B. bassiana*; Lane 2, protein sample isolated from the WT spores; Lane 3, purified BPS8 (200 ng). On top of each lane (Lanes 1 and 2), there were unidentified proteases (bracket-indicated). **(D)** Deletion of *BPS8* significantly decreased rPO1 activity on spores. *B. bassiana* spores of *ΔBPS8*, and WT were incubated with rPPO1. rPO1 activity activated on *ΔBPS8* spores was significantly lower as compared with WT. **(E)** PPO activation by BPS8. Different amounts of BPS8 were incubated with rPPO1 (1 µg) for 10 min, and then PO activity was measured. Please see the Section “Materials and Methods” for the enzyme activity definition for **(A,B,D,E)**. **(F)** Degradation of rPPO1 by BPS8. Supernatants from the incubation buffer containing rPPO1 and different amount of BPS8 for 10 min were loaded for gel analysis, and the Western blot assay indicated that rPPO1 could be degraded by BPS8. Each column represents the mean ± SE estimated from three independent repeats **(A,B,D,E)**. **(G)** BPS8 is a virulence factor to the silkworm larvae and pupae. Survival of silkworm larvae (V-2) following the topic infection with the spores of WT, Δ*BPS8*, and Δ*BPS8:BPS8* (a gene-rescued mutant). Pupae (P-1) were injected with the same number of different spores (1. WT; 2. Δ*BPS8*; 3. Δ*BPS8:BPS8*). The pupae were imaged on day 6 (inset). LC–MS/MS, liquid chromatography–mass/mass spectrometry spectrometry; PO, phenoloxidase; PPO, prophenoloxidase; rPPO1, recombinant PPO1; rPPO2, recombinant PPO2; WT, wild-type.

Phenoloxidase activities were detected when spores were incubated with either rPPO1 or rPPO2 (Figures 6A,B). However, neither binding (Figures 2 and 3) nor PO activities were detected when the bacterial cells of *E. coli* or *Micrococcus* were incubated with rPPO1 or rPPO2. The spores of the insect pathogenic fungus *Metarhizium anisopliae* contain spore-secreted proteases that affect fungal virulence (40). We were curious whether a similar mechanism is involved in activation of PPOs by spore-secreted protease in *B. bassiana*. To examine this possibility, spores were suspended in a binding buffer for 12 h, and a native gelatin zymography analysis was performed. From this we identified one band containing protease activities (Lane 2 in Figure 6C), and a corresponding band in a clean gel (without gelatin but running at the same time) subjected to LC–MS/MS assay identified a member of the peptidase S8 family of proteases (BBA_00319, termed BPS8 for *Beauveria* peptidase S8) based on the eluted fragments (Table S2 in Supplementary Material). The *BPS8* protease is similar to the subtilisin Pr1J of *Metarhizium* that contains an N-terminus I9 family of peptidase inhibitor domain (41). To determine the function of BPS8, the cDNA of this gene was cloned, expressed in *E. coli*, and the purified protein was verified to have protease activity (Lane 3 in Figure 6C). Deletion of *BPS8* gene in *B. bassiana* using a gene-replacement protocol (18) resulted in the disappearance of the protease band in native gelatin zymography (Lane 1 in Figure 6C), and when rPPO1 was incubated with the spores of the WT and gene-deletion mutants (Δ*BPS8*), respectively, the PO activity became significantly lower (*P* < 0.0001) after incubation with the Δ*BPS8* spores than with the WT spores (Figure 6D). The fact that the PO activity was significantly reduced but did not completely disappear in the Δ*BPS8*-treated sample suggested that additional unidentified protease(s) of *B. bassiana* (the bracket-labeled bands in lane in Figure 6C) could also contribute to PPO activation.

We next tested the effect of the protease BPS8 on PPO activity. To do this, purified protease was incubated with rPPO1 at different ratios for 10 min. While cleavage and degradation of rPPO1 were observed for the treatments with higher doses of BPS8 by Western blot assay (Figure 6F), surprisingly PO activity increased at lower doses of protease (Figure 6E). These data suggest that the spore-secreted protease(s) could be co-opted to activate PPOs for insect defense against fungal infection or deployed by pathogen to degrade host-produced PPOs to promote fungal infection. To determine the effect of BPS8 on fungal virulence, we performed insect bioassays using the spores of WT, Δ*BPS8* and gene-rescued mutant (Δ*BPS8*:*BPS8*) for topic infections. The estimation of median lethal time (LT_50_) values revealed that deletion of the gene could significantly (*P* = 0) reduce fungal virulence when comparing the Δ*BPS8* (LT_50_ = 3.5 ± 0.047 days) to the WT (LT_50_ = 3.0 ± 0.061 days). No difference was observed between the WT and gene-rescued mutant (LT_50_ = 3.0 ± 0.013 days) (Figure 6G). When identical numbers of spores were injected into each pupa (P-1) we found that there were fewer conidial spores on the pupal integuments from the gene-rescued mutant (Δ*BPS8:BPS8*) (inset in Figure 6G). Taken together, these data indicate that BPS8 is a virulence factor that can be either utilized by host to activate PPO at low concentration or by fungi to degrade PPO at the same time.

### PPO Effect on Inhibiting Fungal Spore Germination

**Figure 7:**
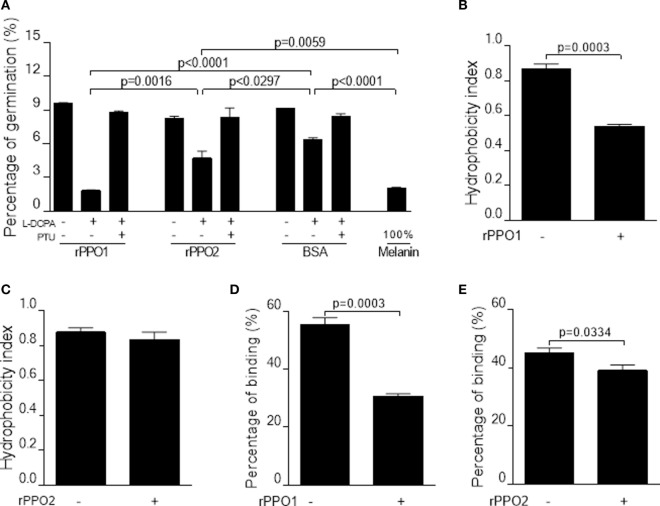
Inhibition on the germination and binding of *B. bassiana* spores after PPO binding. **(A,B)** Pretreatments of fungal spores with PPOs inhibit spore germination. **(A)** Spore germination assay. When rPPO1 or rPPO2 was applied, germination was significantly decreased if the l-DOPA was added to enhance PO-mediated melanization. Significant reduction of spore hydrophobicity was observed when the spores were incubated with rPPO1 **(B)** but not with rPPO2 **(C)**. However, spore adhesion to a hydrophobic surface were significantly impaired after the incubation with either rPPO1 or rPPO2 **(D,E)**. Each column represents the mean ± SE estimated from three independent repeats **(A–E)**. PO, phenoloxidase; PPO, prophenoloxidase; rPPO1, recombinant PPO1; rPPO2, recombinant PPO2.

Melanin, including reactive intermediates of oxygen produced during the process of PO-mediated melanization, is toxic to both pathogens and hosts (42–45). To verify the toxicity of PO on spore viability, we performed spore germination assays using a commercial melanin. The results confirmed that the inhibition of spore germination occurred in a dose-dependent manner (Figure S13 in Supplementary Material). We next examined spore germination rates by incubation of fungal spore with either rPPO1 or rPPO2 with the addition of the PPO substrate l-DOPA and/or the PO inhibitor PTU. While the addition of l-DOPA to BSA (bovine serum albumin) treatment (control) reduced the rate of spore germination as compared with the mock control, an outcome that was likely due to the occurrence of l-DOPA self-oxidization after overnight incubation. Incubation with rPPO1 or rPPO2 could significantly (*P* < 0.05) inhibit spore germination caused by the addition of l-DOPA 12 h post-incubation (Figure 7A). The inhibitory effect of rPPO1 incubation was comparable to the commercial melanin treatment. Not surprisingly, the addition of PO inhibitor PTU could block the PO inhibition of spore germination (Figure 7A). rPPO1 had comparatively a higher inhibitory effect than rPPO2, consistent with the lower PO activity of rPPO2 after being activated (27, 46). When PPO was incubated with hyphae without being activated to induce melanization, there was no obvious influence on the hyphae growth (data not shown). These data demonstrate that the melanization induced by fungal infection can delay the spore germination.

### PPO Pretreatments Impairing Spore Hydrophobicity, Adhesion Activity, and Fungal Virulence

We also performed assays to determine the effect of PPO pretreatments on spore characteristics and insecticidal properties. rPPO1 but not rPPO2 binding could significantly reduce spore hydrophobicity (Figures 7B,C), however pretreatments with both rPPO1 and rPPO2 significantly (*P* < 0.05) reduced spore adhesion ability to a hydrophobic surface (Figures 7D,E).

**Figure 8:**
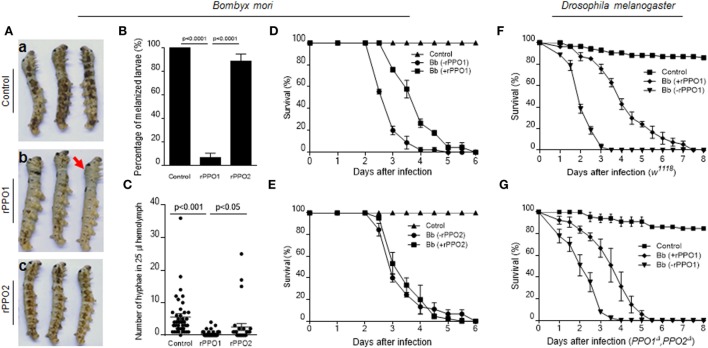
PPO preincubation with spores impairing fungal virulence. **(A,B)** Delayed melanization when *Beauveria bassiana* spores were preincubated with rPPO1 or rPPO2. Topical infection of larvae by spores incubated with nothing but buffer (a; control) or rPPO1 (b) or rPPO2 (c) induced different degree of melanization. Pictures were taken at 40 h post-infection to show different degree of melanization. The arrow in [**(A)**, b] indicates a larva without melanized spots. Melanized larvae induced by rPPO1-incubated spores were significantly lower than the control **(B)**. rPPO2-incubated spores induced less degree of melanization after topical infection. The percentage of melanized larvae were similar to the control but significantly higher than the rPPO1 pretreatment. Each column represents the mean ± SE estimated from three individuals. **(C)** Count of hyphae in hemolymph among different treatments. Low number of hyphae was detected in larvae infected by rPPO1-incubated spores. The data represent the mean ± SE estimated from 30 to 40 individuals. **(D,E)** Survival of silkworm larvae. After being infected with *B. bassiana* spores preincubated with rPPO1 **(D)** or rPPO2 **(E)**, fungal virulence was significantly reduced after the pretreatment with rPPO1 but not rPPO2 as compared with the mock controls (without preincubation, -rPPO1/-rPPO2). **(F,G)** Survival of the WT **(F)** and *PPO* null mutants **(G)** of *Drosophila* adults. After being infected with the spores preincubated with rPPO1, fungal virulence was significantly reduced when assayed against both the WT and mutant flies. Each point represents the mean ± SE estimated from three independent repeats **(D–G)**. PPO, prophenoloxidase; rPPO1, recombinant PPO1; rPPO2, recombinant PPO2; WT, wild type.

Based on these observations, we performed insect bioassays to compare PPO pretreatments of spore on fungal virulence using the silkworm larvae (V-2) and *Drosophila* adults (day 3) as described (18, 28). When spores were preincubated with buffer (control), the larvae melanized significantly at 40 h after topical infection (Figure 8A, a). However, if the spores were preincubated with rPPO1, melanization was reduced significantly (Figures 8A, b; Figure 8B). In contrast, the numbers of melanized larvae and hyphae in hemolymph in the rPPO2 treatment were significantly higher than in the rPPO1 treatment (Figures 8B,C). Topical infection by spores pretreated with rPPO2 induced melanization comparable to the control (Figure 8A, c; Figure 8B). At 40 h, the numbers of hyphae in the hemolymph of larvae infected by rPPO1-treated spores were significantly lower than that in the control (Figure 8C), but the differences were not significant between the control and rPPO2 treatment. It seems that insect PPOs can impair the virulence of *B. bassiana* upon contacting.

Bioassays using spores pretreated with rPPO1, rPPO2 or mock treated were performed on the silkworm larvae (Figures 8D,E). Based on the estimation of LT_50_ values, preincubation of spores with rPPO1 (LT_50_ = 3.933 ± 0.118 days) could significantly (χ^2^ = 35.09, *P* < 0.0001) delay fungal infection compared with the usage of untreated spores (LT_50_ = 2.944 ± 0.081 days) (Figure 8D). No significant delay was detected when rPPO2 was preincubated with spores (χ^2^ = 0.2000, *P* = 0.6547) (Figure 8E). The different effects of rPPO1 and rPPO2 on the virulence of *B. bassiana* spores may be due to the low PO activity in rPPO2 (27, 46). However, in most insects, there are over two or even more PPO genes, and the corresponding PPO proteins can exist at the same time (47). When they work together, the influences should be stronger than each single one.

When the adults of WT *Drosophila* (*w^1118^*) were used for infection, the difference in LT_50_ values between the rPPO1-treated and untreated spores (LT_50_ = 2.136 ± 0.075 and 4.203 ± 0.212 days, respectively) was significant (χ^2^ = 78.63, *P* < 0.0001) (Figure 8F). For adults mutated for both *PPO* genes (*PPO1^Δ^* and *PPO2^Δ^*), LT_50_ value for rPPO1-incubated spores (3.553 ± 0.159 days) was significantly different (χ^2^ = 43.15, *P* < 0.0001) from the LT_50_ value after treatment with unincubated spores (LT_50_ = 2.225 ± 0.119 days) (Figure 8G). These data suggest that PPO-mediated *ex vivo* immunity is likely independent of hemolymph PPO and that pretreatment of fungal spores with PPOs impairs fungal virulence.

## Discussion

Current understandings of insect antimicrobial immune responses and fungal pathogeneses have been obtained principally from observations of *in vivo* interactions between the hosts and pathogens (14, 48, 49). In this study, we present evidence for an *ex vivo* immune-response mechanism mediated by insect PPOs to impede fungal infections, a previously unsuspected strategy for insect survival immediately after ecdysis, a fragile period during the insect life cycle. We found that PPOs in molting fluid could remain bioactive on the surface of newly formed cuticle for an extended period and function to effectively surveil and bind to fungal spores by targeting the cell wall components chitin and β-1,3-glucans. Most interestingly, we found that a specific fungal spore-secreted protease (BPS8) could be utilized to cleave and activate PPOs to produce toxic melanin and inhibit fungal germination and infection if BPS8 is low. PPO is retained in the integuments including cuticles and even epidermal cells (47, 50) and induces melanization when the larvae receive superficial infection by fungi (10, 14). Here we show that when PPO was washed with the molting fluids, the surficial applied conidia could infect the larvae quickly and heavily, by which to induce serious melanization (Figure 1). We further demonstrate that PPOs specifically and effectively decrease fungal germination, hydrophobicity and adhesion activity (Figure 7) to delay the infection (Figure 8).

As type III copper-containing enzymes, insect PPOs play essential roles in both cellular and humoral immune defenses against pathogenic and parasitic infections (15, 47, 51). These proteins catalyze the process of melanization, which usually occurs in insect hemocoels (47, 51). PPO genes have been identified in the genome of different insects, with three copies in the fruit fly *D. melanogaster*, two copies in silkworms and up to nine copies in the mosquito *Anopheles gambiae* (47). In *Drosophila*, PPO1 and PPO2 are activated at different times during the immune response and exhibit varied level of PO activities, thereby conferring different antibacterial activities (15, 27, 52). PPO3 of *Drosophila* is expressed in the lamellocytes and is involved in melanization during the encapsulation process (49). The exact function of these divergent paralogs in other insect is still poorly understood. In this study, we found that insect PPOs (native and recombinantly expressed protein) could bind to the spores of fungal pathogens *via* cell wall components of chitin and β-1,3-glucan. In support of this notion, a previous study demonstrated that *M. sexta* plasma proteins including PPOs could be pulled down by curdlan (53). We found that PPOs can exhibit target selectivity manifest as stronger binding to chitin compared with the cell walls of bacteria and the cell walls of some yeast cells (Figure S4 in Supplementary Material). We postulate that there might be additional unknown factors contributing to this binding selectivity to the spores. A study in mosquitoes indicated that the recruitment of PPOs to the hyphal surfaces of *B. bassiana* was dependent on the melanization regulators TEP1 and CLIPA8 (54), a finding that would support our observation that insect PPOs cannot directly bind to fungal hyphae *in vitro* despite of the presence of similar cell wall components to spores. In contrast to the hydrophobic nature of spores, the blastospores and germinated hyphae of *B. bassiana* as well as yeast cells are hydrophilic, a difference that could be the cause of the reduced PPO binding that we observed, a possibility that we are currently testing. We also found that the pretreatment with insect PPOs could impair the hydrophobicity and adherent properties of spores (Figure 7), leading to reduced fungal virulence (Figure 8). We note that the motifs we identified in rPPO1 and rPPO2 that effectively mediated binding to fungal spore were divergent in sequence and our ongoing work is directed to elucidate the common mechanism of PPO binding.

Upon the detection of pathogenic infection or wound, insect serine proteases such as PAPs, PPAE, and Hayan cleave a conserved peptide bond to convert PPOs into active POs and catalyze melanization (47, 51, 55). Beside the endogenous serine proteases, exogenous proteases like α-chymotrypsin could also activate insect PPOs (56). Insects became heavily melanized when infected by the transgenic mutant of *Metarhizium* overexpressing a fungal protease (43), an observation that supports the idea that PPO activation could also be mediated by a protease from fungal pathogen. In this study, a spore-secreted protease BPS8 of *B. bassiana* was identified that could activate insect PPO (Figure 6), pointing to a strategy that insects might use to exploit the fungus itself to initiate immunity against fungal infection. However, the exact cleavage sites by BPS8 to activate PPOs remain to be determined. We also found that, conversely, rPPO1 could be degraded by BPS8 in a dose-sensitive manner. When the *BPS8* gene was deleted in *B. bassiana*, PO activity could still be observed during the incubation of rPPO1 with the mutant spores (Figure 6D), indicating that additional proteases that contribute to activate or degrade insect PPOs remain to be identified. However, based on this study, we understand that at least BPS8 represents a fungal protein that could be either seized by insect hosts to defend against fungal infection or utilized by fungi to disable host immunity, a vivid example of the balance struck during host–pathogen interactions.

## Author Contributions

JZ, WH, CY, YL, BY, and CW performed and interpreted experiments. PZ and ZZ provided advice and feedback. EL and CW conceived and supervised the project. EL, CW, LD, and JZ wrote the manuscript.

## Conflict of Interest Statement

The authors declare that the research was conducted in the absence of any commercial or financial relationships that could be construed as a potential conflict of interest.
