# Supplementary material for: Prophenoloxidase-Mediated Ex Vivo Immunity to Delay Fungal Infection after Insect Ecdysis
Source: Front Immunol. 2017 Nov 1;8:1445. doi: 10.3389/fimmu.2017.01445 (PMC5671992; doi:10.3389/fimmu.2017.01445)
Supplement: Supplementary file 1 [file Data_Sheet_1.DOCX]

Supplementary Material

**Prophenoloxidase-mediated *ex vivo* immunity to delay fungal infection after insect ecdysis**

Jie Zhang^1,2^, Wuren Huang^1^, Chuanfei Yuan^3,4^，Yuzhen Lu^1^，Bing Yang^1^，Cheng-Yuan Wang^5^, Peng Zhang^5^, Leonard Dobens^6^, Zhen Zou^3^, Chengshu Wang^1,*^, and Erjun Ling^1,*^

^1^Key Laboratory of Insect Developmental and Evolutionary Biology, Institute of Plant Physiology and Ecology, Shanghai Institutes for Biological Sciences, Chinese Academy of Sciences, Shanghai 200032, China

^2^Shanghai Institute of Organic Chemistry Chinese Academy of Sciences, 345 Lingling Road Shanghai 200032

^3^State Key Laboratory of Integrated Management of Pest Insects and Rodents, Institute of Zoology, Chinese Academy of Sciences, Beijing 100101, China

^4^State Key Laboratory of Virology and China Center for Virus Culture Collection, Wuhan Institute of Virology, Chinese Academy of Sciences, Wuhan, 430071, China

^5^National Key Laboratory of Plant Molecular Genetics, Institute of Plant Physiology and Ecology, Shanghai Institutes for Biological Sciences, Chinese Academy of Sciences, Shanghai 200032, China;

^6^School of Biological Sciences, University of Missouri-Kansas City, 5007 Rockhill Road, Kansas City, MO 64110, USA

***Correspondence:**

Chengshu Wang

[cswang@sibs.ac.cn](mailto:cswang@sibs.ac.cn)

Erjun Ling

[ejling@sibs.ac.cn](mailto:ejling@sibs.ac.cn)

**Supplemental Materials and methods**

**Molting fluid residue collection and PO activity assay**

After ecdysis, insect molting fluids become dried within a few minutes ([Zhang et al., 2014](#_ENREF_11)). To collect the remaining proteins left on the surfaces of newly formed cuticles, three silkworm larvae were used at different time points after 4th ecdysis for washing with 200 μl of binding buffer containing PTU. For each time point, several groups od samples were collected and assayed at the same time. The buffer was then centrifuged at 13,000 g at 4℃ for 2 min, and the supernatant was lyophilized at 4℃ and suspended in 20 μl loading buffer and denatured at 95℃ for 5 min for Western blot assay. To detect PO activity in the remaining molting fluid residues, 50 μl of 10 mM dopamine dissolved in 10 mM Tris buffer (pH 7.0) was dropped onto the integument of tape-fixed larvae at different time points after 4th ecdysis in a box with wet papers to keep moisture. The solution was collected 10 min after the treatment. The baseline solution contained PTU to inhibit oxidization of dopamine. The samples were centrifuged at 13,000 g for 1 min, and the optical density of supernatant was recorded at 490 nm (OD_490_) as the readout of PO activity.

**Purification, collection and expression of PPOs from different insects**

Native PPOs were purified from the plasma of both *Bombyx mori* (BmPPO) and *Helicoverpa armigera* (HaPPO) larvae as described ([Jiang et al., 1997](#_ENREF_4)). To collect the plasma PPOs from *Drosophila*, ten larvae of mutant fly *PPO1^∆^* or *PPO2^∆^* ([Binggeli et al., 2014](#_ENREF_1)) were washed and dried on towel paper. Hemolymph was released into 20 μl binding buffer after teasing the integument using two forceps. The samples were then centrifuged at 3,000 g at 4℃ for 5 min. The supernatant containing either PPO1 or PPO2 were used for further analysis. Recombinant PPO1 (rPPO1), rPPO1-GFP and PPO2 (rPPO2) of *Drosophila* were expressed in *E. coli*, and purified ([Li et al., 2012](#_ENREF_5); [Yang et al., 2013](#_ENREF_10)). All PPO proteins were stored at -80℃ after determining the concentration.

**Identification of PPO binding motif**

After the pre-incubations with rPPO1 or rPPO2, spores of *B. bassiana* were washed three times, re-suspended in binding buffer containing 0.1 μg/μl Trypsin (Sigma) and incubated at 37℃ for 4 h. The spores were then washed three times using the washing buffer containing 500 mM NaCl, and then suspended in 4% SDS to elute remaining peptides that might bind to spores. The samples were applied for LC-MS/MS assay to determine the peptide fragments as described ([Shao et al., 2012](#_ENREF_8)). To verify the affinity binding peptides derived from PPO1 and PPO2, the fragments were fused with GFP by PCR with the primer pairs (Table S1) at either N- or C- terminus. The fused proteins were expressed and purified for binding examinations by Western blot or under fluorescence microscope.

**Molecular docking analysis**

The structures of two *Drosophila* PPOs were modeled with *Manduca sexta* PPO crystal structure (PDB ID 3HHS) as a homologous template as described before ([Li et al., 2009](#_ENREF_6)). The receptor (PPO) and ligand files (chitin and β-1,3-glucan) were prepared with AutoDock Tools and the PRODRG ([Schuttelkopf and van Aalten, 2004](#_ENREF_7)), and then each PPO was retained for ligand docking with AutoDock Vina ([Trott and Olson, 2010](#_ENREF_9)). The volume of the cavity of the modeled PPO was calculated by using the CCP4 suite ([Collaborative Computational Project, 1994](#_ENREF_2)). Images were prepared using Pymol version 1.30 (DeLano Scientific).

**Competition assay**

(1) For the competition assay using a typical carbohydrate sugar, rPPO1 or rPPO2 (1 μg) was incubated with approximately 1◊10^7^ spores containing 200 μg sugar (Sigma) in binding buffer (50 mM Tris, 100 mM NaCl, pH 8.0) with a total volume of 50-μl. Mixtures were incubated by rotating at 4℃ for 15 min and then centrifuged at 13,000 g at 4℃ for 2 min. The spore pellets were washed three times by resuspension in 100 μl washing buffer (100 mM Tris, 500 mM NaCl, pH 8.0) followed by centrifugation (13,000 g at 4℃ for 2 min). The flow-through and spores were suspended in SDS loading buffer and boiled for 10 min for Western blot assay. Unless otherwise indicated, after binding assay using rPPO1 or rPPO2, approximately 8% or 16% of flow-through and elution solution treated as described above were loaded for each lane respectively in this work.

(2) Competition among rPPO1 or rPPO2 and rPPO1-GFP was conducted as follows. rPPO1 (0.015 μM) or rPPO2 (0.015 μM) and rPPO1-GFP (0.015 μM) were mixed with 1◊10^7^ spores in a 50-μl system and incubated by rotating at 4℃ for 15 min. The mixtures were centrifuged to obtain the follow-through solution. The spores were washed and proteins were eluted for Western blot assay as described above.

(3) Competition by peptidoglycan on rPPO1 and rPPO2 binding to spores was performed as described here. Peptidoglycan (4 μg) from Gram-negative (G-) bacteria *E. coli 0111:B4* (PGN-EB; InvivoGen) and Gram-positive (G+) bacteria Staphylococcus aureus (PGN-SA; InvivoGen) were separately incubated with 2 μg rPPO1 or rPPO2 for 15 min at room temperature, and then half of the mixture was removed and added to 1◊10^7^ spores in a 50-μl system, and the mixture was incubated by rotating at 4℃ for 15 min. The mixtures were centrifuged to obtain the flow-through solution, and the spores were washed as described above and proteins were eluted for Western blot assay.

(4) Competition of chitin and β-1,3-glucan on rPPO1 and rPPO2 binding to spores was conducted as follows. Because chitin and β-1,3-glucan are granules and are not suitable to be incubated with spores, to perform this assay approximately 2 μg rPPO1 or rPPO2 was incubated with 2 mg chitin and β-1,3-glucan respectively in a 100-μl system by rotating at 4℃ for 15 min. Two aliquots from the mixture were centrifuged (13,000 g at 4℃ for 2 min). For one aliquot, chitin or β-1,3-glucan was washed and eluted as described above (including the flow-through) for Western blot assays. For another aliquot, following centrifugation, flow-through recovered was incubated with 1◊10^7^ spores in a 50-μl system and incubated by rotating at 4℃ for 15 min. After incubation, the flow-through and elution of spores were prepared as described above for Western blot assay.

**References**

Binggeli, O., Neyen, C., Poidevin, M., and Lemaitre, B. (2014). Prophenoloxidase activation is required for survival to microbial infections in *Drosophila*. *PLoS Pathog.* 10(5)**,** e1004067. doi: 10.1371/journal.ppat.1004067.

Collaborative Computational Project (1994). The CCP4 suite: programs for protein crystallography. *Acta Crystallogr D Biol Crystallogr.* 50(Pt 5)**,** 760-763. doi: 10.1107/s0907444994003112.

Huang, W., Shang, Y., Chen, P., Cen, K., and Wang, C. (2015). Basic leucine zipper (bZIP) domain transcription factor MBZ1 regulates cell wall integrity, spore adherence, and virulence in *Metarhizium robertsii*. *J Biol Chem.* 290(13)**,** 8218-8231. doi: 10.1074/jbc.M114.630939.

Jiang, H., Wang, Y., Ma, C., and Kanost, M.R. (1997). Subunit composition of pro-phenol oxidase from *Manduca sexta*: molecular cloning of subunit ProPO-P1. *Insect Biochem Mol Biol.* 27(10)**,** 835-850. doi: S0965-1748(97)00066-0 [pii].

Li, X., Ma, M., Liu, F., Chen, Y., Lu, A., Ling, Q.-Z., et al. (2012). Properties of *Drosophila melanogaster* prophenoloxidases expressed in *Escherichia coli*. *Dev Comp Immunol.* 36(4)**,** 648-656. doi: 10.1016/j.dci.2011.03.034.

Li, Y., Wang, Y., Jiang, H., and Deng, J. (2009). Crystal structure of *Manduca sexta* prophenoloxidase provides insights into the mechanism of type 3 copper enzymes. *Proc Natl Acad Sci U S A.* 106(40)**,** 17002-17006.

Schuttelkopf, A.W., and van Aalten, D.M. (2004). PRODRG: a tool for high-throughput crystallography of protein-ligand complexes. *Acta Crystallogr D Biol Crystallogr.* 60(Pt 8)**,** 1355-1363. doi: 10.1107/s0907444904011679.

Shao, Q., Yang, B., Xu, Q., Li, X., Lu, Z., Wang, C., et al. (2012). Hindgut innate immunity and regulation of fecal microbiota through melanization in insects. *J Biol Chem.* 287(17)**,** 14270-14279. doi: 10.1074/jbc.M112.354548.

Trott, O., and Olson, A.J. (2010). AutoDock Vina: improving the speed and accuracy of docking with a new scoring function, efficient optimization, and multithreading. *J Comput Chem.* 31(2)**,** 455-461. doi: 10.1002/jcc.21334.

Yang, B., Lu, A., Peng, Q., Ling, Q.Z., and Ling, E. (2013). Activity of fusion prophenoloxidase-GFP and its potential applications for innate immunity study. *PLoS One* 8(5)**,** e64106. doi: 10.1371/journal.pone.0064106.

Zhang, J., Lu, A., Kong, L., Zhang, Q., and Ling, E. (2014). Functional analysis of insect molting fluid proteins on the protection and regulation of ecdysis. *J Biol Chem.* 289(52)**,** 35891-35906. doi: 10.1074/jbc.M114.599597.

**Supplemental Figures and Figure legends:**

**
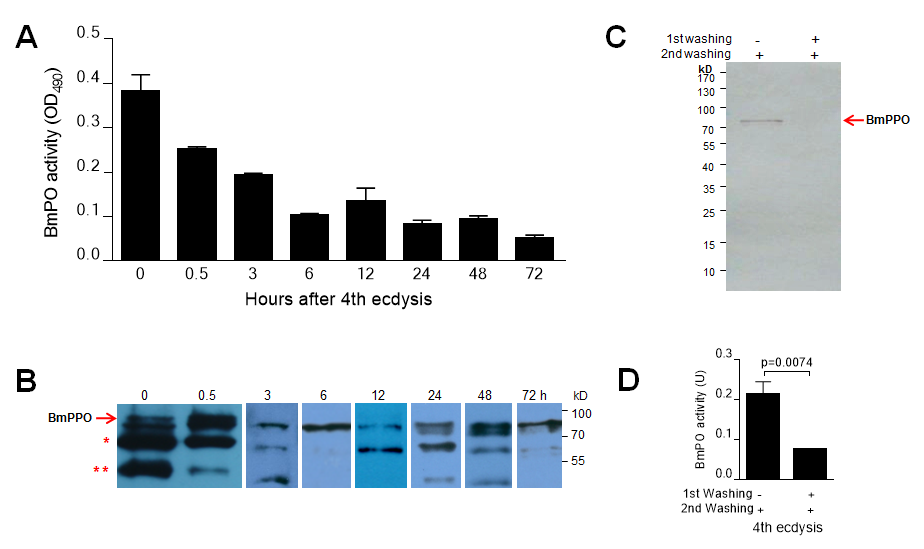
**

**Supplementary Figure 1. Duration of bioactive molting fluid proteins left on the newly formed insect cuticles.** (A) BmPO activities remaining in the molting fluids. Dopamine (200 μl) was dropped to the surface of each tape-fixed larva at different time points after ecdysis and incubated for 10 min at room temperature in a wet box. In one group, some saturated PTU was added to dopamine solution to inhibit PO activity as baseline. After being centrifuged to remove debris, optical density was read and treated as PO activity. (B) Detection of BmPPO in molting fluids after ecdysis. Three larvae were washed using binding buffer at the indicated time point after ecdysis. Several groups of samples were collected. One result was selected for each time point. The arrow points to BmPPO. The asterisks indicate the degraded fragments of BmPPO. (C, D) Detection of BmPPO after washing the newly-molted larvae or not. The newly-molted silkworm larvae were washed for the first time (-) or not (+) using sterile water 2 h post 4th ecdysis. At 15 min, the remained molting fluids were washed for the second time as described above either for BmPPO (arrow-pointed) detection (C) or PO activity (D) assay.


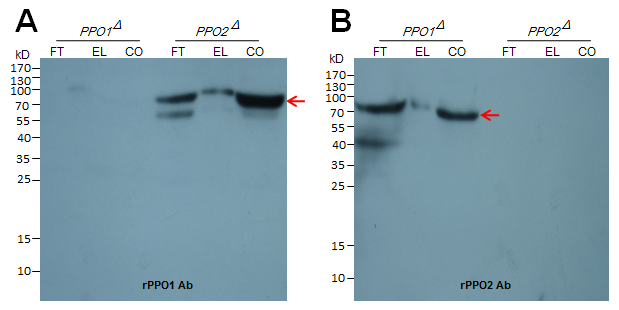


**Supplementary Figure 2. Verification of *Drosophila* plasma PPO1 and PPO2 binding to fungal spores.** Plasma PPO1 and PPO2 were collected from 10 larvae of *Drosophila* mutant *PPO2^Δ^* or *PPO1^Δ^* into 20 μl of binding buffer. After centrifugation, the plasma samples were incubated with *B. bassiana* spores at 4℃ for 15 min. PPO1 (A) and PPO2 (B) bound to *B. bassiana* spores were eluted after being heated in 1×SDS loading buffer. Each membrane was detected using different antibody as indicated. The arrows point to PPO1 and PPO2, respectively. FT, flow through; EL, elution; CO, control.


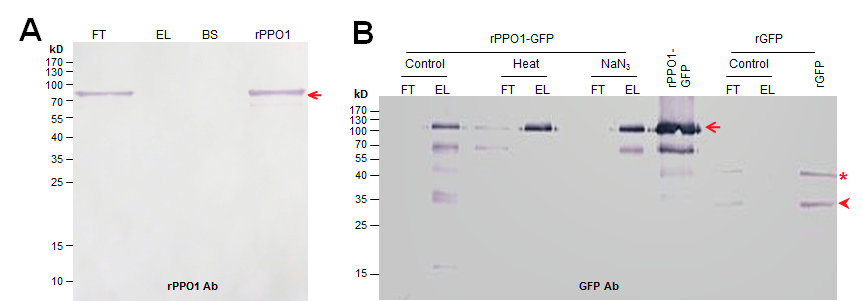


**Supplementary Figure 3. Verification of rPPO1-GFP binding to *B. bassiana* spores.** (A) A Western blot assay showing that rPPO1 did not bind to blastospores. The arrow points to rPPO1. FT, flow-through; EL, elution; BS, blastospores of *B. bassiana*. (B) A Western blot assay showing the binding of rPPO1-GFP but not rGFP to *B. bassiana* spores, and spores that were killed by heating or NaN_3_ treatment. The arrow points to rPPO1-GFP and the arrowhead points to rGFP. The asterisk indicates likely a dimer of rGFP in this purification.


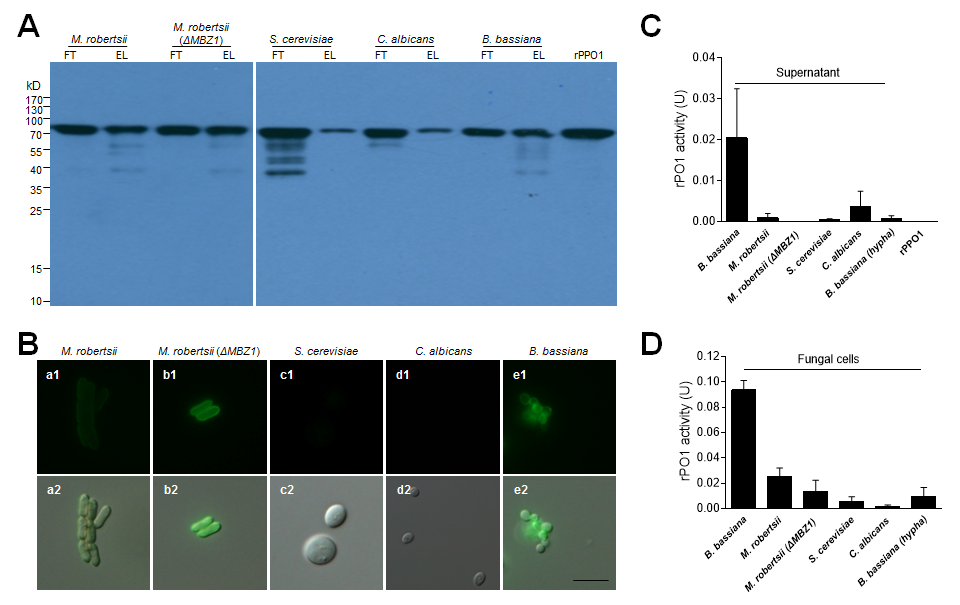


**Supplementary Figure 4. Binding activity of rPPO1 to the spores of different fungal species.** (A) Western blot analysis of rPPO1 binding to different fungal spores. Spores of different fungal species such as *S. cerevisiae*, *C. albicans*, *M. robertsii* and *M. robertsii* mutant *(ΔMBZ1)* were incubated with the same amount of rPPO1 at room temperature for 15 min. In addition to *B. bassiana* spores, rPPO1 could also bind to spores of different fungal species. However, the binding abilities of rPPO1 varied among different fungal spores. FT, flow-through; EL, elution. (B) Microscopic examination of rPPO1-GFP binding to the cells of different fungi. rPPO1-GFP can bind to the spores of wild type *M. robertsii* (a1, a2), *M. robertsii* mutant (*ΔMBZ1*) (b1, b2) that contain higher amount of chitin in spores ([Huang et al., 2015](#_ENREF_3)), and *B. bassiana* (e1, e2). rPPO1-GFP barely bound to the cells of yeast (c1, c2) and *C. albicans* (d1, d2). The top panels were taken under the green filter while the bottom panels were merged with those imaged using differential interference contrast (DIC). (C, D) rPPO1 activities in the flow-through (C) and on each type of fungal spores (D) were assayed by using dopamine. Fungal cells were removed before optical density reading. The flow-through from *B. bassiana* spores had a higher rPO1 activity (C). After binding to fungal spores, rPPO1 on the *B. bassiana* was also higher than the others (D). Each column represents the mean ± S.E. estimated from three independent experiments. Bar: 5 μm.


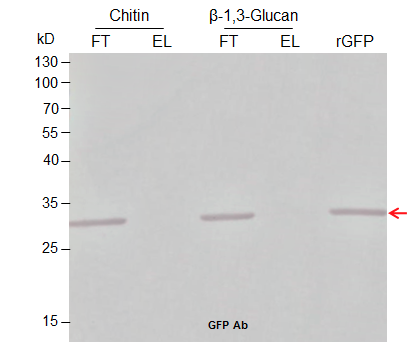


**Supplementary Figure 5. GFP fails to bind to chitin and β-1, 3-glucan.** As were PPO proteins shown in Figure 4A-4F, rGFP (1 μg) was incubated with chitin and β-1, 3-glucan respectively. The flow-through and bound proteins eluted from either chitin or β-1, 3-glucan were prepared for Western blot analysis. rGFP can be detected in the flow-through but not in the elution solution of chitin or β-1, 3-glucan. The arrow indicates rGFP. Approximately 8% of eluted proteins from chitin or β-1, 3-glucan and 8% of flow-through proteins were loaded for each lane. FT, flow-through; EL, elution; rGFP, control protein without incubation (0.08 μg).


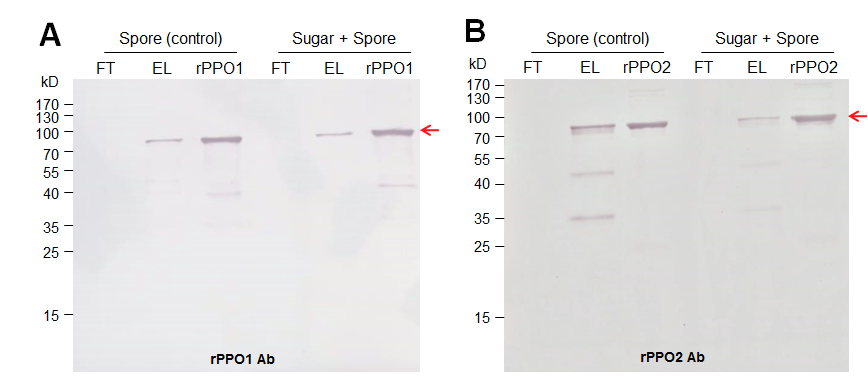


**Supplementary Figure 6. Co-incubation with sucrose did not affect rPPO1 or rPPO2 to bind to spores.** Approximately 500 μg of sugar was co-incubated with rPPO1 (A; 1 μg) or rPPO2 (B; 1 μg) and spores. As a control, rPPO1 (A; 1 μg) or rPPO2 (B; 1 μg) was incubated with spores without sucrose. Sucrose did not interfere with binding of either rPPO1 or rPPO2 to spores. The arrows indicate rPPO1 (A) and rPPO2 (B) respectively. The lanes labeled as rPPO1 (A; 0.08 μg) and rPPO2 (B; 0.16 μg) are control protein without binding. (B) FT, flow-through; EL, elution.


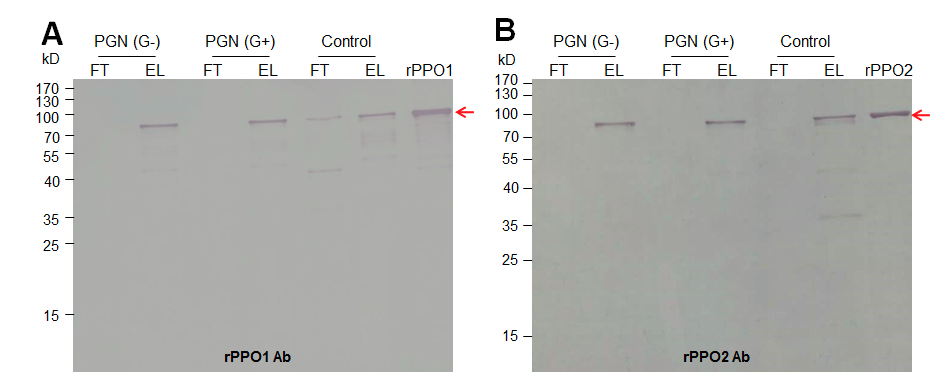


**Supplementary Figure 7. Peptidoglycan (PGN) from Gram-positive or Gram-negative bacteria did not affect rPPO1 or rPPO2 binding to spores.** Peptidoglycan (2 μg) from Gram-positive (G+) and Gram-negative (G-) were pre-incubated with 1 μg of rPPO1 or rPPO2 respectively for 15 min followed by addition of spores. As a control, rPPO1 (A) or rPPO2 (B) was incubated with spores without peptidoglycan. Peptidoglycan from Gram-positive and Gram-negative bacteria did not interfere with rPPO1 or rPPO2 binding to spores, respectively. The arrows indicate rPPO1 (A) and rPPO2 (B), respectively. The lanes labeled as rPPO1 (A; 0.08 μg) and rPPO2 (B; 0.16 μg) are control protein without binding. FT, flow-through; EL, elution.


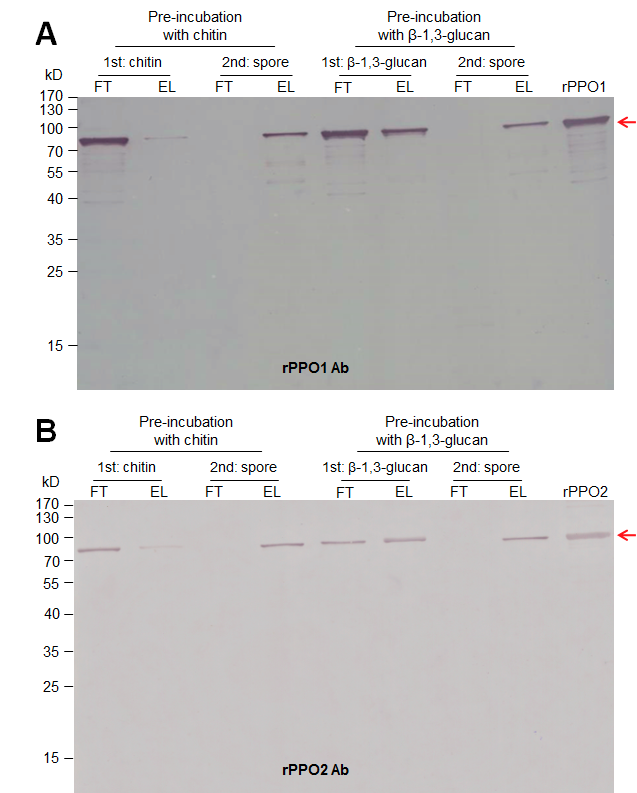


**Supplementary Figure 8. rPPO1 and rPPO2 pre-incubated with chitin or β-1, 3-glucan retain the ability to bind spores.** Chitin and β-1, 3-glucan used in this work are granules, and they cannot be separated from spores after co-incubation. Therefore, rPPO1 and rPPO2 were separately pre-incubated with chitin and β-1, 3-glucan, and then the flow-through containing free rPPO1 or rPPO2 was collected for the spore binding assay (see the Supplemental Materials and methods for detail). 1st: chitin and 1st: β-1, 3-glucan: Half of the pre-incubation mixture was centrifuged for chitin and β-1, 3-glucan respectively for eluting the bound proteins (EL). In this case, approximately 10% for rPPO1 treatment (20% for rPPO2 treatment) of eluted proteins from chitin or β-1, 3-glucan (EL) and 10% for rPPO1 treatment (20% for rPPO2 treatment) of flow-through (FT) proteins were loaded for each lane. 2nd: spore: The other half of the mixture was centrifuged for the follow-through that contained free rPPO1 or rPPO2, and then was added with spores for incubation. After that, spores and flow-through were collected as above. The arrows indicate rPPO1 (A) and rPPO2 (B) respectively. The lanes labeled as rPPO1 (A; 0.08 μg) and rPPO2 (B; 0.16 μg) are control proteins without binding. FT, flow-through; EL, elution.


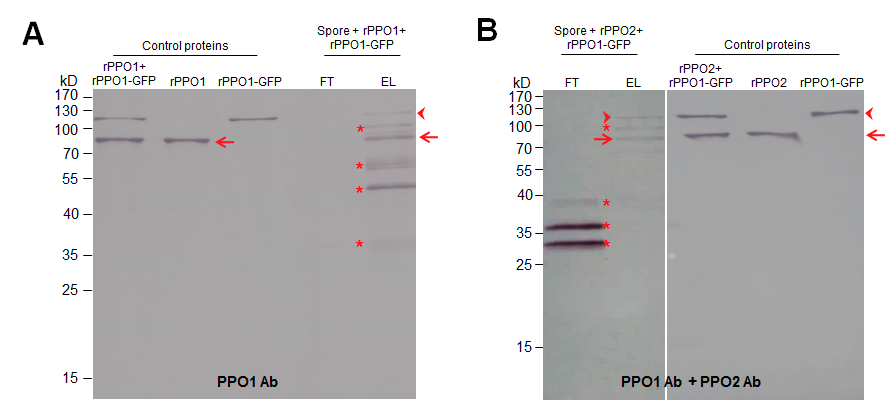


**Supplementary Figure 9. Co-incubation rPPO1 or rPPO2 with rPPO1-GFP did not affect spore binding.** rPPO2-GFP has very low PO activity, thus rPPO1-GFP was used for a competition assay against rPPO2. The same concentrations of rPPO1 or rPPO2 and rPPO1-GFP were co-incubated with spores. rPPO1 (A) or rPPO2 (B) and rPPO1-GFP did not affect each other’s binding. The arrowheads indicate rPPO1-GFP, and the arrows point to rPPO1 or rPPO2 as indicated above each panel. The asterisks indicate the degraded bands from either rPPO1 or rPPO2 or rPPO1-GFP. In (A), approximately 0.08 μg of rPPO1 and 0.08 μg of rPPO1-GFP was loaded for each lane either separately or collectively as indicated. In (B), approximately 0.16 μg of rPPO2 and 0.08 μg of rPPO1-GFP was loaded for each lane either separately or collectively as indicated. FT, flow-through; EL, elution.


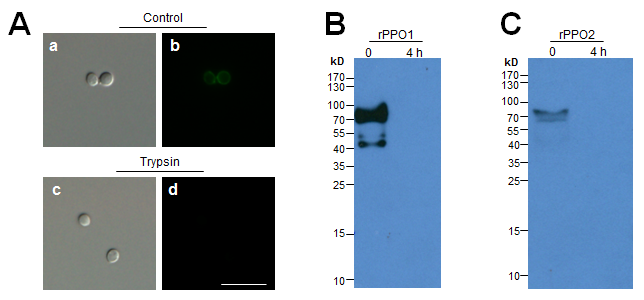


**Supplementary Figure 10. Degradation of rPPO1 and rPPO2 bound to *B. bassiana* spores using trypsin.** (A) rPPO1-GFP bound to spores could be degraded by trypsin. rPPO1-GFP was incubated with *B. bassiana* spores (a, b). The spores were suspended in a buffer containing trypsin for 0.5 h, the fluorescence signal was lost (c, d). Bar: 5 μm. (B, C) rPPO1 was incubated with *B. bassiana* spores. After washing, the spores were suspended in buffer containing trypsin for 0 and 4 h. At each time point, the spores were separated by centrifuge and mixed with 1 × SDS loading buffer and heated for elution. After being incubated with trypsin for 4 h, the bound rPPO1 and rPPO2 were degraded and could not be detected on a 12% SDS-PAGE gel.


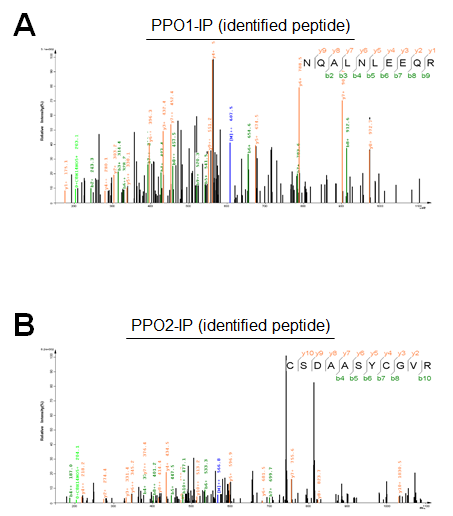


**Supplementary Figure 11. Identification of the fragments of rPPO1 (A) and PPO2 (B) that bind to *B. bassiana* spores through LC-MS/MS analysis.** rPPO1 or rPPO2 bound to *B. bassiana* spores was digested with trypsin. The spores were washed three times using washing buffer and then eluted for LC-MS/MS spectrum assay. The fragments of PPO1 (A) and PPO2 (B) that were eluted from spores were named as PPO1-IP and PPO2-IP, respectively.


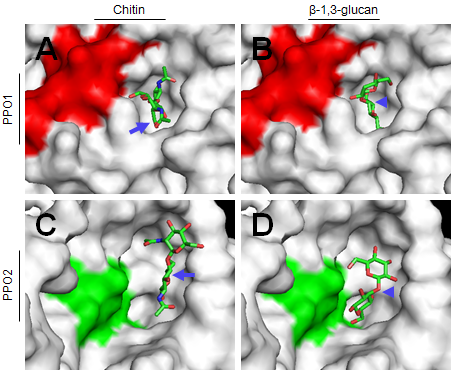


**Supplementary Figure 12. *In silico* docking analysis.** The affinity binding fragments of *Drosophila* PPO1 (^518^NQALNLEEQR^527^) and PPO2 (^622^CSDAASYCGVR^632^) were identified after being eluted from *B. bassiana* spores were labeled in red (A, B) and green (C, D), respectively. Ligands of chitin (A, C; Arrow-indicated) and β-1,3-glucan (B, D; Arrowhead-indicated) were depicted with a stick representation, respectively. Chitin and β-1,3-glucan are predicted to dock into the cavities partly composed of the above fragments that were identified after affinity binding to *B. bassiana* spores.


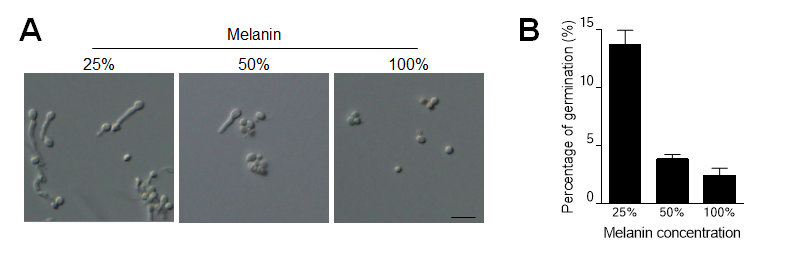


**Supplementary Figure 13. Inhibition of fungal spore germination by melanin.** (A) Microscopic examination of the germination of *B. bassiana* spores after being co-cultured with different concentrations of melanin for 12 h. (B) Statistical estimation of spore germination rates by addition of different concentrations of melanin for 12 h.

| **Table S1 Oligonucleotide primers used in this study** | |  |
| --- | --- | --- |
|  |  |  |
| **Primer** | **Sequence (5' to 3')** | **Usage** |
| BPS8 KO UF | ATCTGCAGGCGTTTCAGCCATATCAGCA | For construction of gene disruption vector pBAR-MBZ1 |
| BPS8 KO UR | CGGATCCCACTGCTCTCATGTGGCATC |  |
| BPS8 KO DF | GCTCTAGATTGCCTCCAAGTCGTATGGT |  |
| BPS8 KO DR | CGAGCTCCGGCACTGACAACCATGATT |  |
|  |  |  |
| BPS8 F | CCATGGTTGGCTTCAAGAGCTT | For recombinant protein expression in E.coli |
| BPS8 R | GAATTCTTAATGATGATGATGATGATGCTGGTTGCCGTTGTAAGAG |  |
|  |  |  |
| PPO1-IP-GFP NF | CCATGGCCAATCAGGCTTTGAATCTGGAGGAGCAGCGAATGGTGAGCAAGGGCGAG | For fusion expression with GFP at N or C terminus in E. coli |
| PPO1-IP-GFP NR | GAATTCTCAGTGATGGTGATGGTGATGCTTGTACAGCTCGTC |  |
| GFP-PPO1-IP CF | CCATGGCCCATCACCATCACCATCACATGGTGAGCAAGGGCGAG |  |
| GFP-PPO1-IP CR | GAATTCTCATCGCTGCTCCTCCAGATTCAAAGCCTGATTCTTGTACAGCTCGTCCAT |  |
| PPO2-IP-GFP NF | CCATGGCCTGCAGCGATGCCGCTTCCTACTGTGGCGTCCGTATGGTGAGCAAGGGCGAG |  |
| PPO2-IP-GFP NR | GAATTCTCAGTGATGGTGATGGTGATGCTTGTACAGCTCGTC |  |
| GFP-PPO2-IP CF | CCATGGCCCATCACCATCACCATCACATGGTGAGCAAGGGC |  |
| GFP-PPO2-IP CR | GAATTCTCAACGGACGCCACAGTAGGAAGCGGCATCGCTGCACTTGTACAGCTCGTC |  |

| **Table S2 BPS8 identification by LC-MS/MS.** | | | | | | |  |  |  |  |  |  |  |
| --- | --- | --- | --- | --- | --- | --- | --- | --- | --- | --- | --- | --- | --- |
|  |  |  |  |  |  |  |  |  |  |  |  |  |  |
|  |  |  |  |  |  |  |  |  |  |  |  |  |  |
| **Reference** | **PepCount** | **UniquePepCount** | **CoverPercent** | **MW** | **PI** | **IdentifiedName** | |  |  |  |  |  |  |
| **File, Scan(s)** | **Sequence** | **MH+** | **Diff(MH+)** | **Charge** | **Rank** | **XC** | **DeltaCn** | **Sp** | **RSp** | **Ions** | **PI** | **GroupCount** | **ProteinCount** |
|  | 8 | 3 | 14.94% | 41564.94 | 5.77 | BBA_00319 |  |  |  |  |  |  |  |
| Protein_A,8106 | K.VFSGPTADTSVILDGYNWAVNDIVAK.R | 2754.0436 | 0.3456 | 2 | 1 | 3.4084 | 0.5857 | 1199.5 | 1 | 22\|50 | 3.93 | 1 | 1 |
| Protein_A,8112 | K.VFSGPTADTSVILDGYNWAVNDIVAK.R | 2754.0436 | 0.0886 | 2 | 1 | 4.1724 | 0.6486 | 718 | 1 | 20\|50 | 3.93 | 1 | 1 |
| Protein_A,8146 | K.VFSGPTADTSVILDGYNWAVNDIVAK.R | 2754.0436 | 1.2086 | 2 | 1 | 3.1001 | 0.5295 | 705.9 | 1 | 20\|50 | 3.93 | 1 | 1 |
| Protein_A,8162 | K.VFSGPTADTSVILDGYNWAVNDIVAK.R | 2754.0436 | 1.2206 | 2 | 1 | 2.7645 | 0.5116 | 1306.7 | 1 | 21\|50 | 3.93 | 1 | 1 |
| Protein_A,5846 | R.APQAITVAAVNSEWSR.W | 1700.877 | 0.393 | 2 | 1 | 3.406 | 0.5445 | 1043.7 | 1 | 17\|30 | 6.05 | 1 | 1 |
| Protein_A,5890 | R.APQAITVAAVNSEWSR.W | 1700.877 | 0.392 | 2 | 1 | 3.9333 | 0.5517 | 1818.4 | 1 | 21\|30 | 6.05 | 1 | 1 |
| Protein_A,6297 | R.SVINLSLGGGASTAFDR.A | 1665.8294 | 1.3874 | 2 | 1 | 2.8781 | 0.4937 | 621.3 | 1 | 16\|32 | 5.55 | 1 | 1 |
| Protein_A,6333 | R.SVINLSLGGGASTAFDR.A | 1665.8294 | 0.1464 | 2 | 1 | 3.6595 | 0.5772 | 904.9 | 1 | 18\|32 | 5.55 | 1 | 1 |
